# Movement simulations reveal both memory and social information drive individual foraging site fidelity in gannets

**DOI:** 10.1186/s40462-026-00656-8

**Published:** 2026-05-14

**Authors:** Christopher J. Pollock, Jude V. Lane, Ruth Jeavons, Victoria Warwick-Evans, Robert W. Furness, Tim G. Benton, Keith C. Hamer

**Affiliations:** 1 School of Biology, University of Leeds, Leeds, LS2 9JT UK; 2 UK Centre for Ecology & Hydrology, Bush Estate, Penicuik, EH26 0QB UK; 3 RSPB Centre for Conservation Science, The Lodge, Sandy, SG19 2DL UK; 4 Atlantic58, UHI North, West & Hebrides, The Old Weaving Shed, Stornoway, HS2 0XR UK; 5 Ecosystems Team, British Antarctic Survey, Cambridge, CB3 OET UK; 6 SLR Consulting, 93 South Woodside Road, Glasgow, G20 6NT UK

**Keywords:** Northern gannet, Seabirds, Individual foraging site fidelity, Individual-based model, Movement simulations

## Abstract

**Background:**

Alongside their direct perceptions of prey, Northern gannets (*Morus bassanus*) likely derive foraging cues from memory of previously successful foraging areas (private information), and from conspecifics (public information). Together these mechanisms are likely to underpin emergent Individual foraging site fidelity (IFSF) patterns, characterised by individual consistency in foraging area use. Where observation methods are inadequate in helping us determine IFSF properties, simulation experiments can help decipher the implicit foraging strategies present in an organism’s movements.

**Methods:**

We began by developing a movement simulation model to simulate spatially explicit foraging trips of chick-rearing gannets at Bass Rock, capturing direct perception of prey only. Once confidence in the foundational model was achieved, we made modifications to the model to incorporate various implementations of public and private information use in foraging strategies. The model outputs from simulations experiments, encompassing a wide range of complexity, were compared to several empirically derived patterns at the individual and population level, to gain insight into which mechanisms may be driving IFSF patterns and the potential consequences on a population’s space use.

**Results:**

Patterns observed in standalone foraging trips were well represented by our movement simulations, permitting further developments to include public and private information use. Mechanisms using private information, namely memory based reuse of previously successful departure directions, were the primary drivers of IFSF. Incorporating social information refined these patterns, improving alignment with prey distributions and enhancing realism at the population-level, irrespective of the private information employed. However, mechanisms that best reproduced emergent individual and population level spatial patterns were associated with reduced foraging efficiency, inferred from longer trip durations and greater travel distances, indicating a trade-off between spatial segregation and short-term efficiency.

**Conclusion:**

Our results support the idea that IFSF can emerge from hierarchical foraging strategies in which long-term private information structures broad-scale space use, while social information modulates fine-scale movements. By linking behavioural mechanisms to emergent movement patterns in a spatially explicit environment, our study illustrates how individual-based models can move beyond description to generate mechanistic and predictive insights into animal movement, improving our ability to anticipate responses to environmental change.

**Supplementary Information:**

The online version contains supplementary material available at 10.1186/s40462-026-00656-8.

## Background

Animal foraging behaviour in heterogenous and dynamic environments often differs consistently among phenotypically similar individuals, where space use variation reflects how individuals acquire and use information [1, 2]. Such individual variation is particularly relevant in colonial systems, where many conspecifics exploit overlapping resource landscapes. Individual foraging site fidelity (IFSF) is a form of individual specialisation which is characterised by consistent use of foraging areas by an individual that are significantly narrower than that of its given population. Found to persist over long time periods in a range of species [3–5], IFSF is thought to contribute to reduction in intraspecific competition, an effect that may be especially pronounced in colonial species and has been shown to result in higher foraging efficiency [6, 7] and influence reproductive success [5].

Many species at higher trophic levels face heterogeneously distributed and ephemeral prey, coupled with incomplete knowledge of its whereabouts and both intra- and interspecific competition. Thus, behaviour which distils information from various cues which can offer fitness advantages will be favoured by natural selection [8]. In an information rich environment with numerous potential cues, individuals may acquire and weight different information sources in distinct ways, generating substantial phenotypic variation in space use [9]. Typically overlooked in favour of testing for effects of factors such as species or sex, where this has been sufficiently characterised [10], research focus has shifted to determining the form and magnitude of individual variation and its consequences.

Seabirds provide a particularly informative system for which to examine these processes, as they act as central-place foragers during the breeding season. Repeatedly exploiting foraging areas that are simultaneously accessible to many conspecifics creates high levels of competition for non-uniform and hierarchically clustered resources [11] likely driving the complex foraging movements they exhibit. Sinuous and relatively slow-moving area-restricted search (ARS) behaviour is thought to occur in response to patches with higher prey density [12], which are typically linked by faster, more linear movements between patches and to and from the colony. Across successive trips it is thought that seabirds may be faithful to a directional arc, departing the colony at a similar bearing, known as the “trap line” strategy [13] which can contribute to IFSF by promoting consistent use of particular localities in the foraging landscape. However, if foraging success declines, they may switch areas employing a win-stay, lose-shift (WSLS) strategy [14, 15]. As central place foragers are expected to minimise time spent travelling and searching for prey [16], particularly under the energetic constraints of breeding. Therefore, mechanisms that reduce overlap with competitors may confer substantial advantages.

Mechanisms may draw on private information, acquired through individual experience and memory. Theory predicts memory to be advantageous when resources exhibit temporal persistence, suggesting fitness benefits at moderate spatiotemporal complexity [17]. Predictability in marine systems is often associated with physical features, such as shelf edges, frontal zones, and upwellings, that persist over varying time scales [18] and result in areas of enhanced productivity. Northern gannets (*Morus bassanus*, hereafter “gannets”) preferentially forage in spatially predictable, seasonally persistent frontal zones rather than more ephemeral contemporaneous fronts [19]. Pettex et al. [20] show that gannets’ foraging trips are more or less linear to and from foraging areas, and repeatability across trips [21] further suggest anticipation of prey locations based on prior experience.

Alongside private information, colonially nesting seabirds exploit public information derived from conspecifics [22]. Cues on the whereabouts of prey may be provided from the perception of other birds foraging at sea, known as local enhancement [23]. For example, Cape gannets (*Morus capensis*) alter their foraging trip trajectory in response to external stimuli such as boats and other marine predators [24]. However, theory predicts that the benefits of local enhancement depend strongly on the competitive context, with gains expected only when competition costs remain low [25, 26]. In colonial systems where many individuals converge on the same areas, public information may also function to avoid areas of high conspecific density, rather than solely to locate prey. In the North Sea, where dense shoaling fish are relatively uncommon, observations of gannets at sea find that feeding aggregations are small and short-lived [27]. This suggests that an avoidance mechanism using public information may be particularly relevant.

Taken together, the combined use of private and public information to locate heterogeneously distributed resources, is likely to be key in driving the emergence of IFSF in colonial, central-place foraging seabirds [28]. Wherein, private information accrued over broader time periods helps in relocating particular areas and public information is invoked at finer spatiotemporal scales to refine prey localisation during foraging trips. Empirical testing of how individuals use private and social information is possible [e.g., 29, 30], but poses many challenges especially in marine systems. Studies of Cape gannets indicate use of both social cues and memory [28, 31] but have been unable to determine to what extent behavioural decisions are based on these cues. In lieu of empirical studies, simulation experiments can help us unravel the potential mechanisms contributing to the emergence of empirically detectable patterns, such as IFSF, when said mechanisms are posited in various ways to encompass a wide range of complexity.

The aim of this study was to determine whether the observed IFSF in gannets during the chick-rearing period results from the use of private information, public information, or a combination of both. To this end, we developed a foundational, spatially explicit IBM of gannets at the world’s largest breeding colony of this species, Bass Rock, Scotland (~ 150,000 breeding adults [32]) with the initial requirement of simulating realistic foraging trips of gannets during the chick-rearing period. We then implemented various mechanisms using private (e.g., long- or short-term memory) and public (e.g., local enhancement or avoiding intraspecific competition) information in simulation experiments in an attempt to decipher the underlying foraging strategies driving IFSF patterns. To achieve this, we used pattern-oriented modelling (POM) [33, 34], whereby several empirically derived patterns from an extensive GPS tracking dataset were used to guide development, parameterisation and evaluation at both the individual and population level to provide insight into IFSF patterns and their influence on foraging efficiency.

Individual-based models (IBM) provide a suitable framework for addressing this question by; (i) allowing simulation of an organism’s complex decisions based on its needs, opportunities and environment [35]; (ii) having the potential to include interactions whereby individuals can influence and be influenced by others [e.g., 36], and; (iii) allowing the incorporation of memory where we understand it to be unlikely that individuals have perfect information about their environment [e.g., 37]. What emerges from such IBMs at the population-level is the consequence of the complex interactions between individuals and their environments. Relevant applications of IBMs include investigating the memory of previously visited areas in reproducing realistic home ranges in marine mammals [38], and similar investigations to ours into the drivers of IFSF in gannets by Wakefield et al. [39], albeit in a spatially implicit environment. As recommended by Aarts et al. [40] developing species specific IBMs and fitting these models to spatially explicit data [e.g., 41, 42] can help reveal the most likely behavioural mechanisms underlying the observed patterns.

## Methods

Here we provide summarised information on the telemetry data, a description of the movement simulations and how we parameterised and evaluated them. We then describe the modifications made to our foundational model to incorporate mechanisms using private and public information, and how we compared the model outputs against empirical equivalents to evaluate IFSF patterns in our simulation experiments. A detailed model description following the standardised overview, design concepts, details (ODD) protocol [43, 44], further biological background and an expanded results section is included in the supplemental material.

### Telemetry data and usage

**Fig. 1:**
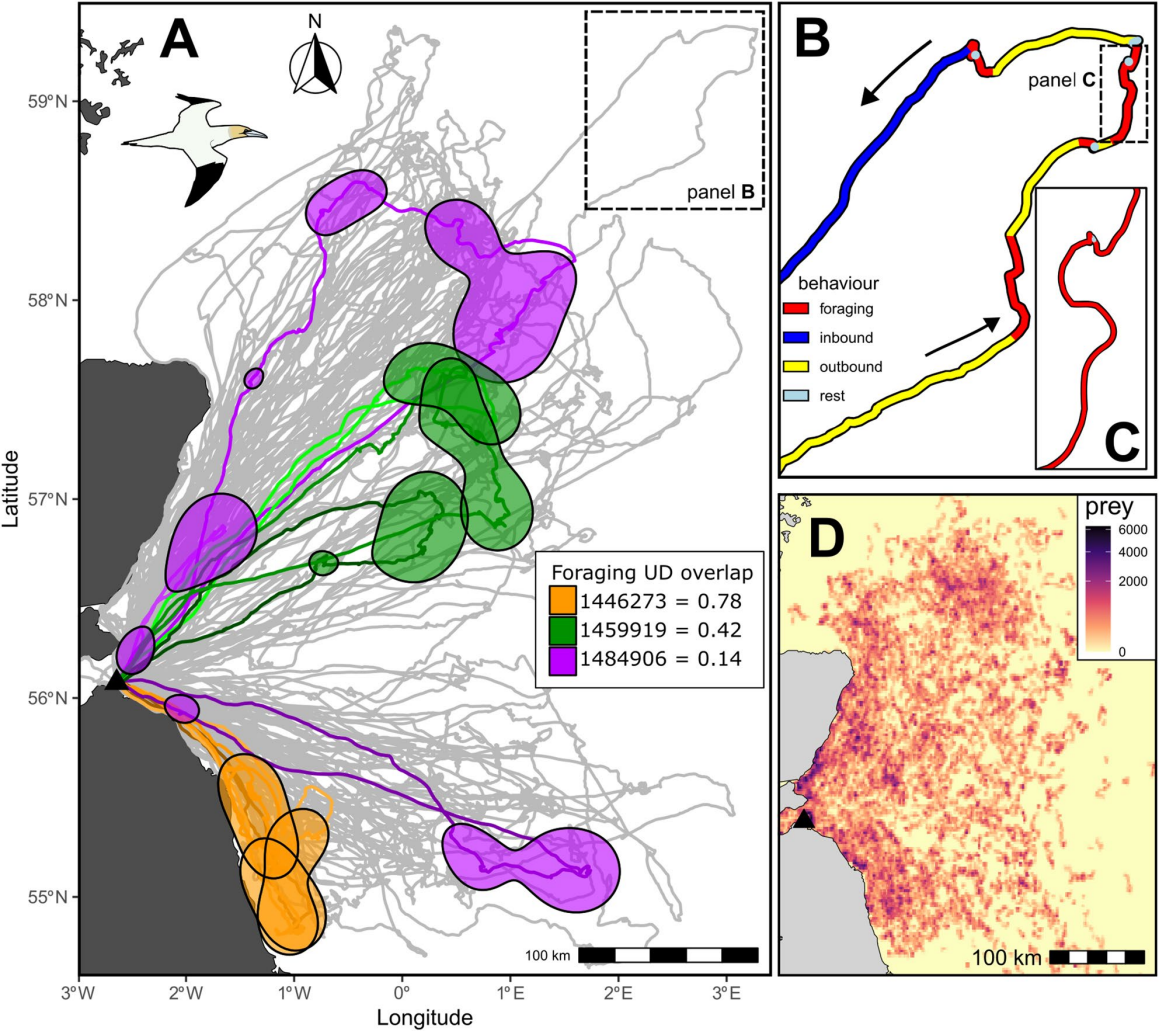
The different patterns used in our study: (**A**) shows empirical tracking data from gannets at Bass Rock (black triangle) to display patterns associated with individual foraging site fidelity (IFSF). Grey paths show 114 trips from 29 individuals (year = 2015), with orange, green and purple paths displaying three consecutive trips from three individuals, with kernels of the same colour representing 50% utilisation distributions of foraging locations only. Bhattacharyya’s affinity (BA) scores provide quantification of the mean foraging utilisation distribution (UD) overlap within each individual by taking a mean of the three pairwise combinations. (**B**) focuses on a specific track to show categorisation of different movement modes, where (**C**) is inset to highlight tortuosity of foraging ARS movement. (**D**) displays the prey density layer (arbitrary units) used in simulations which is derived from foraging locations of gannets from empirical tracking data

Movement data from chick-rearing adult gannets were obtained in 2011, 2012, 2015 and 2016 using GPS loggers (igotU-GT600, Mobile Action Technology, Taipei, Taiwan). Loggers were attached to the upper side of the central tail feathers with tape and set to record locations at 2-minute intervals. For more information on tagging procedures see Wakefield et al. [4] and Lane et al. [45]. The dataset comprised 504 trips from 118 individuals and was implemented to (i) assign approximate prey density to cells in the seascape being modelled from where birds foraged according to empirical data (Figs. 1C, S3), (ii) extract key patterns of foraging trips to guide model development, parameterisation and evaluation, and (iii) classify behaviours associated with IFSF for evaluation of our simulation experiments.

### Modelling

All IBM modelling was implemented in NetLogo version 6.1.1 [46] with code provided on GitHub (https://github.com/ChrPol/gannet_movement_IBM). All processing and analysis of model outputs and empirical data was conducted in R v4.1.0 [47].

### Movement simulations of foraging trips

Our foundational model, a spatially explicit IBM of chick-rearing gannets at Bass Rock, was designed to capture realistic movements of foraging trips that are a consequence of an individual’s decisions, based upon their immediate environment and foraging success during a given trip. The main entity in our model is adult chick-rearing gannets which interact with their environment while on foraging trips, where land cells are actively avoided [48]. Individuals are characterised by their location, step length (i.e., speed), turning angle, the movement mode they are currently in (outbound/inbound travel, ARS, rest), and how much food intake there has been on the trip so far. The simulated landscape is the area of the North Sea (381,888 km^2^) representing Bass Rock’s breeding gannets’ foraging range. Prey is distributed heterogeneously in 2 × 2 km cells (Fig. 1C) and was determined by the amount of time gannets spent foraging in these areas from empirical data. Data on gannet prey availability is not available at the required spatiotemporal resolution, and Warwick-Evans et al. [49] show increased foraging behaviour in areas where gannets spend more time. Prey can be depleted during each day according to how many successful foraging attempts were recorded, allowing for increasing competition as a day progressed. Prey was reset to initial levels at the beginning of each new day.

The model’s discrete two-minute timesteps reflect the temporal resolution of the empirical data (“virtual ecologist” approach [50]). The model was run for a total of 91 days, representing the length of the chick-rearing period. In each timestep gannets on a foraging trip check their behaviour and update their behavioural tally counters for respective movement modes. Then, according to their location, current stomach contents, and density of prey on their current patch, they enact a behaviour by withdrawing a step length and turning angle characterised for the corresponding movement mode, or switch to a different movement mode. Individuals can cycle between various movement modes while on a foraging trip (Figure S4) including outbound travel, ARS and rest, before beginning inbound travel back to the colony once a threshold amount of prey has been required (Fig. 1B). The individual then remains at the colony until the next trip.

Gannets exhibit an approximation of a type II functional response [51] wherein the transition probability of commencing ARS and of consequently catching fish within ARS, increases linearly with prey density up to a point where it remains constant after a defined prey density is exceeded (Figure S5). The broad scale arc of a trip emerges from a logistic relationship between food intake for the current trip influencing the difference in bearing (0-180°) from the initial one taken from the colony (Figure S7). Initial departure angle for this model was based on individuals selecting the bearing of an area of high prey density, with no requirement to reach it. Trips were randomly assigned with equal chance of clockwise or anticlockwise travel direction at the beginning of the trip.

#### Parameterisation, sensitivity analysis, and evaluation

Parameter values used in this model were derived from literature or empirical data where possible (Table S4). For parameters where values were not available, we employed a series of parameter estimation procedures, guided by pattern-oriented modelling using three steps in the following order: (i) We assessed fine-scale movements by comparing step length and turning angle distribution outputs from 100 simulated trips run with different stochasticity coefficients to empirical equivalents. (ii) Three interacting parameters for the transition probability of commencing ARS/feeding were simulated at various levels, then outputs of trip durations (hrs) were compared to empirical data using root mean square error (RMSE) scores to determine the best parameter combination. (iii) The logistic relationship between food intake and adjustment to the initial bearing of the colony, determining an individual’s heading related to the colony, was varied across three levels to determine the broad scale shape of the trip in respect to how elliptical or linear the emerging shapes of trips were.

We performed a local sensitivity analysis by running singular, successive perturbations of + 10% and − 10% on a subset of important model parameters. For each perturbation trip durations (hrs) from 1000 trips belonging to 100 individuals were withdrawn, and the difference in mean trip duration was expressed as a percentage difference from that of the baseline trip duration from a model run with no parameter perturbations. Final evaluation of the model was achieved through side-by-side comparisons of simulated and observed trips into their shape and form, and for activity budgets, with further observations of population patterns by plotting 100 trips to detect any emergence of population-level patterns.

### Individual foraging site fidelity simulation experiments

#### Resource localisation mechanisms

We devised four respective mechanisms for different uses of private and public information, including a null model with no information use. Pairwise combination resulted in 16 different simulation conditions (Table 1) with the aim of capturing a wide range of potential mechanisms for information use in the foraging strategies of gannets. For further biological background to IFSF patterns see S2.2, and for justification for and descriptions of the different mechanisms see S5.

**Table 1:** Simulation conditions for individual foraging site fidelity (IFSF) experiments obtained through different combinations of resource localisation mechanisms using public and private information

	***Public information***				
***Private information***		**No social interaction (1)** No influence from conspecifics	**Local enhancement (2)** Attraction to nearby conspecifics engaged in ARS movement	**Competition (3)** Do not begin foraging in an area occupied by several conspecifics	**Enhancement and competition (4)** Uptake of enhancement opportunities only when not in an occupied area
	**No memory (A)** Random departure angle due east (5-140°) from the colony for each trip	**Null model** **A1**	**Public** **A2**	**Public** **A3**	**Public** **A4**
	**Long-term memory (B)** Consistent departure angle determined prior to chick-rearing period, no switching	**Private** **B1**	**Combination** **B2**	**Combination** **B3**	**Combination** **B4**
	**Short-term memory/WSLS (C)** Consistent departure angle, unless three previous trips are increasing in duration resulting in new random departure angle	**Private** **C1**	**Combination** **C2**	**Combination** **C3**	**Combination** **C4**
	**Combined memory (D)** Prior determined departure angles (x3) which can be switched between as in C	**Private** **D1**	**Combination** **D2**	**Combination** **D3**	**Combination** **D4**

#### Simulation specifications

We made several modifications to the foundational model to allow for the use of private and/or public information. Departure angle choice for simulations with no private information was chosen at random between the bounds of 5-140°, reflecting the general direction of travel inferred from empirical data while avoiding the implication of memory from previous trips. For simulations using private information a list containing trip departure angles and their respective trip durations, as a proxy for foraging efficiency, was stored by each individual acting as memory with different forms of selection depending on the mechanisms being simulated (Table 1). Some simulations incorporating long-term memory (B & D in Table 1) required a “burn-in” period of ten trips in random departure angles (5-140^o^) to allow for exploratory trips prior to the simulated period of interest to represent knowledge being carried into the chick-rearing season which had been gained previously. Combination D specifically assumes individuals have developed knowledge of reliable productive sites through prior exploration [52], functioning analogously to a cognitive map. Although prey availability is ephemeral, the persistence of oceanographic features that aggregate prey [18] provides a basis for this site directed behaviour mechanism. Departure angle was chosen as the memorised movement to act upon as it would allow for emergence of potential navigation error [20] and furthermore, permits individuals to exploit foraging opportunities as they arise on the outbound trip thus foraging trips are as short as possible and in concordance with optimal foraging theory [53].

In simulations invoking public information, interactions with conspecifics were considered once > 10 km from the colony. Conspecifics could be detected in a 270^o^ field of vision from their current heading up to 10 km away [31]. We modelled local enhancement (2, Table 1) through detection of any conspecifics exhibiting ARS behaviour in a 10 km range. If detected, individuals had a 10% chance per timestep of pursuing a given conspecific’s witnessed foraging location at 5–10 km, increasing to 20% below 5 km. Upon reaching the location where the foraging conspecific was first observed, outbound behaviour and orientation prior to local enhancement was resumed. To represent competition (3, Table 1) any conspecifics, regardless of movement mode, were detected up to 5 km away and if there were more than two other gannets detected, individuals could not begin ARS movement. When combined (4, Table 1), uptake of local enhancement opportunities was only possible when below the determined threshold competition level.

For each simulation experiment we ran the model with 1,000 gannet individuals as modelling the entire population was beyond our computational means. For processes in which population size is likely to have a strong influence, such as interactions with conspecifics, where empirical evidence is lacking and difficult to obtain, the model was heuristically parameterised so that the public information in question was exhibiting a detectable, but not overwhelming, effect on individuals (Figure S13). All gannets began the simulation at the colony and departed at random over the ensuing 24 h. The main outputs of the model were the behaviour, trip number, and coordinates recorded at each timestep for a subset of 100 individuals.

#### Evaluation and analyses

We quantified IFSF at two temporal scales using simulated tracks derived from either three or six consecutive trips per individual. In both cases, tracks were drawn from 100 individuals, yielding a total of 300 or 600 simulated trips, respectively. Model outputs were evaluated against GPS tracks by extracting the maximum number of individuals possible from the data set for either three (*n* individuals = 98, *n* trips = 294) or six (*n* individuals = 33, *n* trips = 198) consecutive trips (Figure S22). Since most foraging trips in our complete empirical dataset were overnight (83%, Table S9), we did not distinguish between same-day trips and those spanning one night or more to investigate differential use of accrued information. To evaluate how well each model reproduced observed levels of IFSF three individual-level patterns were quantified including:

(i) Bhattacharyya’s affinity (BA) was calculated as a measure of individual consistency in the use of foraging areas following Wakefield et al. [4] by measuring the overlap of 50% kernel densities of foraging locations for three or six consecutive trips. A score of zero indicates no overlap, and 1 indicates perfect overlap. We also created a null distribution of BA scores using a randomisation procedure. If the null hypothesis was rejected, we could infer that the observed IFSF differed significantly from a random assignment of bird ID to trips (see S3.3 for further description). To evaluate model performance, we calculated the Mean Absolute Error (MAE) to quantify the magnitude of the difference between simulated and observed BA scores per individual. Finally, a Kolmogorov-Smirnov (KS) test was used to compare the simulated and observed distributions of BA scores, determining whether the model successfully replicated the overall spread and shape of individual consistency observed in the empirical data. If *P* > 0.05 this is interpreted as inability to distinguish a model’s output from the observed data at a 95% confidence level.
(ii) Repeatability of departure angle was calculated using three consecutive trips using circular ANOVAs [54], with higher values indicating within-individual variance being lower than between-group.
(iii) Repeatability of trip duration and its standard error were calculated along with a p-value testing the null hypothesis that within-individual variation is equal to between-group variation using the R package ‘rptR’ [55].

A further four population-level patterns were used to assess which simulations best exploited the available prey distribution and which were most efficient:

(iv) To assess how well simulated foraging areas aligned with the putative distribution of prey in the model we directly compared grids of simulated foraging locations to that of the prey using Schoener’s D index. This index ranges from zero (no overlap) to 1 (complete overlap). To get a sense of the general and core spatial footprints, and enable better visual comparison, we also calculated the utilisation distribution overlap index (UDOI [56]), between 95% and 50% UDs from simulation foraging locations to that of the UDs used to infer the prey density distribution in the model. Values typically range from zero, indicating low overlap to one indicting high overlap, but can exceed this if the two UDs being compared are nonuniformly distributed and have a high degree of overlap.
(v) Average daylight trip duration (hrs) was extracted as a proxy for population-level foraging efficiency [57].
(vi) Average furthest distance from the colony (km) was also used for the same purposes, where foraging further afield could indicate higher competition or individuals which are less effective at finding prey [57].
(vii) Finally, we compared the distribution of the population’s departure bearings with that of the empirical data using KS tests to see if this pattern was well represented.

## Results

### Realistic simulations of foraging trips

The resulting step lengths for two-minute time steps were 1.82 ± 0.34 km and 1.84 ± 0.35 km for outbound and inbound travel, respectively, and 9.02 ± 0.44 km for ARS movement, reflecting empirical data well (Figure S8). Coefficients influencing the stochasticity of turning angles resulted in distributions which were comparable to those seen empirically for respective movement modes (Figure S9). The parameterisation procedure for interacting parameters involved in the transition probability of commencing ARS/feeding resulted in selection of the following parameters: prey.density = “low”; prey.detect = 0.125; ThresholdARS = 20, as although it didn’t have the lowest RMSE score when compared to empirical trip durations (Table S5), this combination resulted in the best alignment with the median and interquartile ranges of empirical data (Figure S11). Different parameter values relating to the steepness of the logistic relationship between food-intake and adjustment to the initial bearing resulted in linearity indexes for trips that were consistently within the bounds of those seen in empirical data. Due to being considerably more linear in some years that others (Figure S12), we opted for the intermediate (“normal”) logistic curve going forwards (Figure S7). The sensitivity analysis indicated that model trip durations were most sensitive to the parameters associated with the transition probability of commencing ARS/feeding procedure. Principally, the parameter which dictates the chance of detecting prey, followed by food intake requirement (Figure S14).

**Fig. 2:**
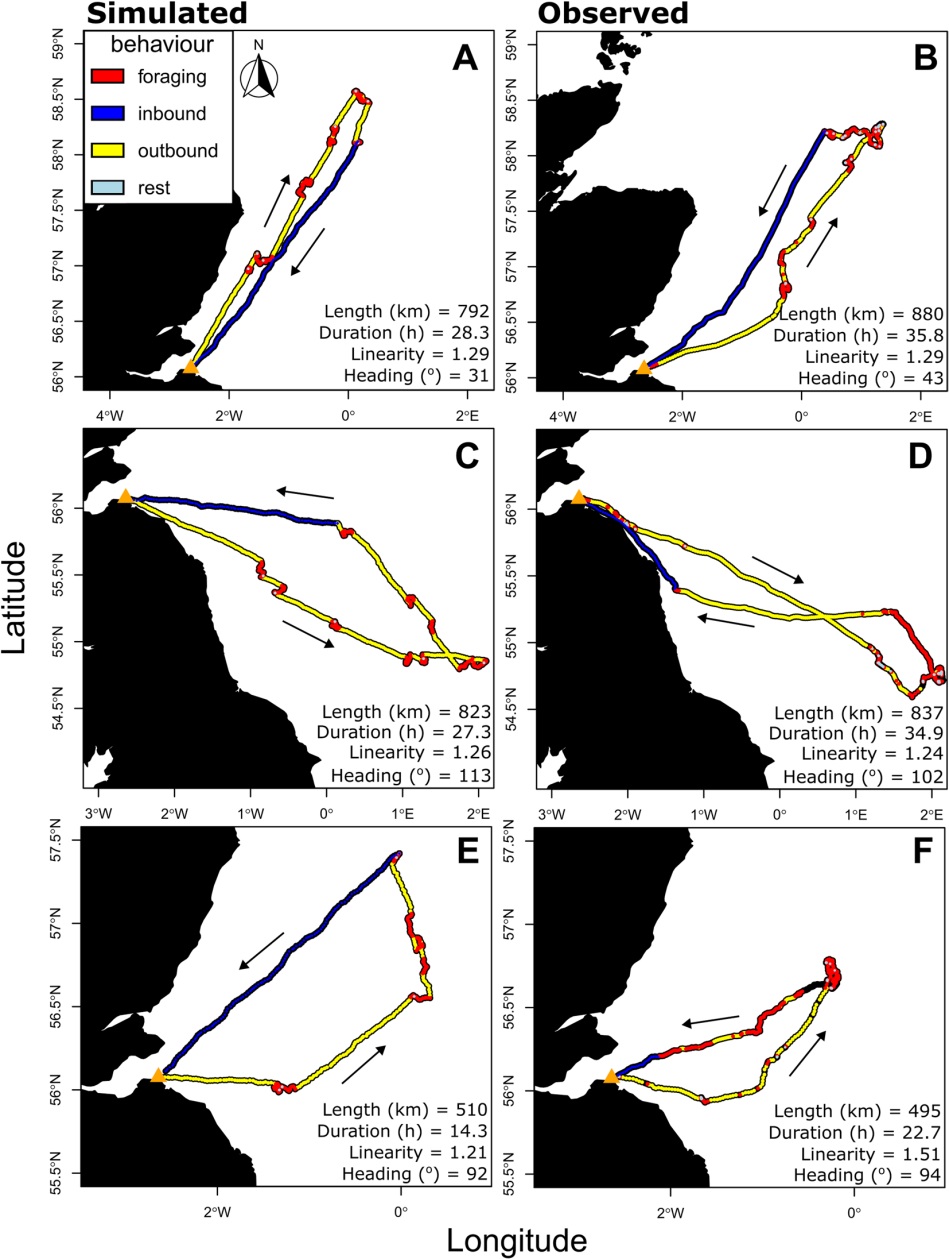
Side-by-side comparison of three simulated foraging trips in left column (**A**, **C** & **E**), with three similar trips from empirical data in the right column (**B**, **D** & **F**) with trip metrics inset in respective panels. Colours indicate different movement modes: yellow = outbound travel, red = ARS, light blue = rest, dark blue = inbound travel. Inbound travel in observed tracks was determined through visual inspection of tracks for the last area of ARS prior to returning to the colony. The orange triangle indicates the location of Bass Rock

From visual evaluation of the model, we have captured many attributes observed in empirical foraging tracks, both in terms of size and shape, with one drawback being the relatively straight travel between ARS zones (Fig. 2) which is an artefact of the bearing adjustment being a product of food intake which can only occur when in ARS. Inspections of plots at population-level show the emergence of population-level space use comparable to that seen empirically (Figure S15).

### Individual foraging site fidelity simulation experiments

**Table 2:** Sample size and IFSF-related trip metrics for observed data across years of data collection, subdivided into datasets used for three and six consecutive trip analyses

Number of consecutive trips per individual analysed	Metric	All years combined	2011	2012	2015	2016
3	*N individuals*	*98*	*21*	*30*	*25*	*22*
	Individual consistency in use of foraging areas using Bhattacharyya’s affinity (BA) score	0.47 (0.12, 0.99) ***	0.56 (0.18, 0.98) ***	0.45 (0.18, 0.81) ***	0.47 (0.12, 0.77) ***	0.41 (0.13, 0.72) ***
	Repeatability of departure angle (R)	0.54 ± 0.06	0.39 ± 0.14	0.70 ± 0.08	0.69 ± 0.09	0.28 ± 0.14
	Daylight trip duration (hrs)	16.4 ± 8.3	15.3 ± 8.21	16.4 ± 9.8	18.6 ± 6.8	14.7 ± 7.3
	Maximum distance from colony (km)	198 ± 103	141 ± 98	199 ± 99	242 ± 99	203 ± 105
6	*N individuals*	*33*	*13*	*9*	*6*	*5*
	Individual consistency in use of foraging areas using Bhattacharyya’s affinity (BA) score	0.53 (0.26, 0.99) ***	0.64 (0.32, 0.99) ***	0.48 (0.33, 0.81) ***	0.39 (0.26, 0.64) ***	0.49 (0.37, 0.66) ***
	Repeatability of departure angle (R)	0.68 ± 0.07	0.68 ± 0.1	0.84 ± 0.08	0.66 ± 0.17	0.53 ± 0.22
	Daylight trip duration (hrs)	13.8 ± 7.1	13.7 ± 7.1	12.8 ± 6.4	17.7 ± 7.2	11.2 ± 6.7
	Maximum distance from colony (km)	162 ± 97	126 ± 80	170 ± 87	237 ± 96	154 ± 107

### Three consecutive trips

**Fig. 3:**
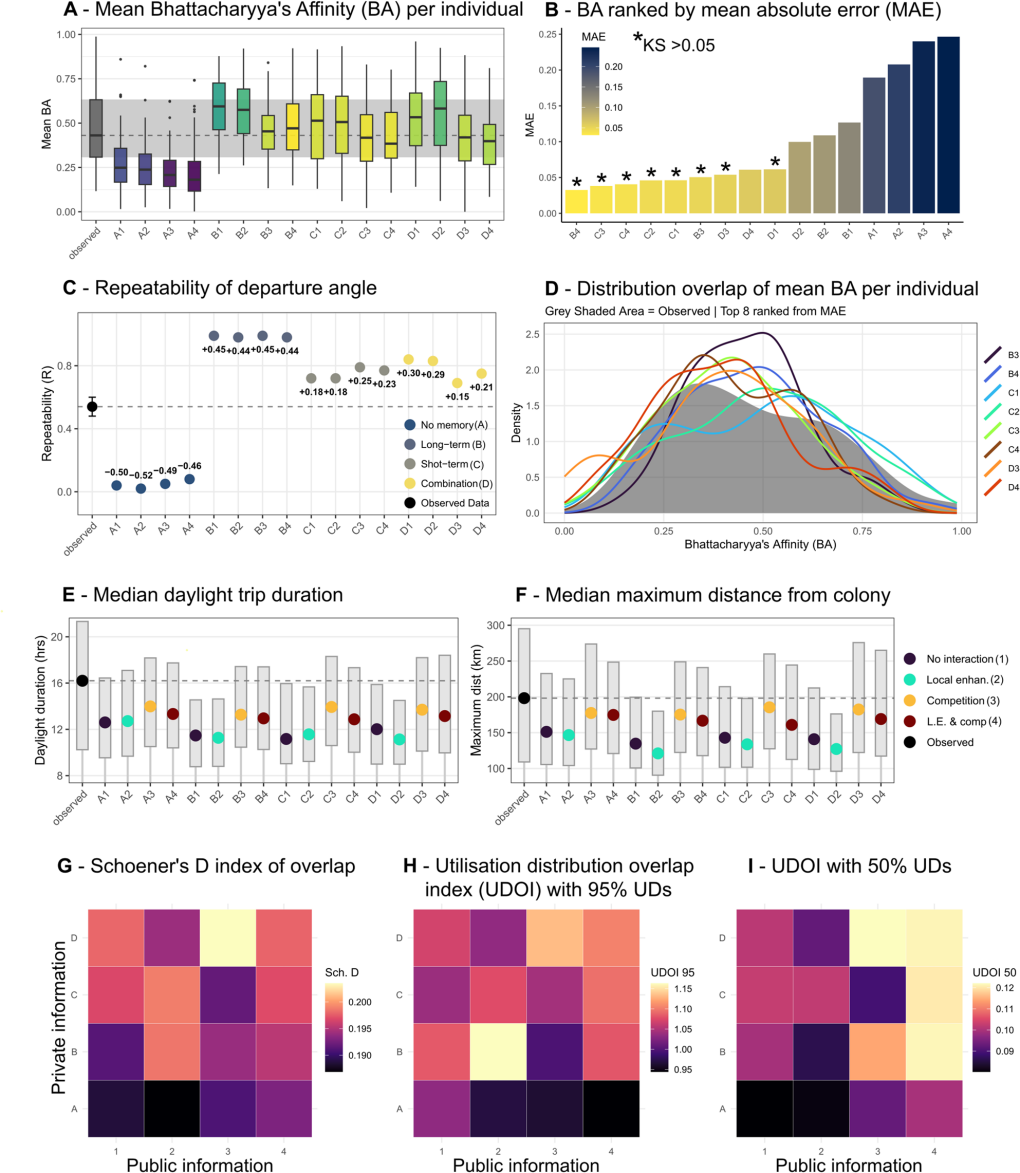
Comparison of simulated movement models against observed data for three consecutive foraging trips per individual. (**A**-**B**) Model performance based on Bhattacharyya’s Affinity (BA) and ranked by Mean Absolute Error (MAE), where lower MAE indicates a better fit to observed data. Horizontal grey band in (**A**) represent the interquartile range of mean BA per individual for observed data (*n* = 98, simulated *n* = 100). Bars marked with an asterisk in (**B**) show Kolmogorov-Smirnov (KS) test results for density distributions of simulation vs. observed BA where the null hypothesis that the distribution of the simulation is identical to the observed distribution is accepted. (**C**) Repeatability of departure angles with simulation points coloured by different memory mechanisms and black for observed data. (**D**) Density distribution of mean BA per individual for the top 8 performing models according to MAE (see **B**) compared to observed data. (**E**-**F**) Comparison of median daylight trip duration and maximum distance from the colony with colours of points representing different social interaction mechanisms and black for observed data. (**G**-**I**) Spatial overlap metrics comparing analysed trips to the putative prey distribution including Schoener’s D and Utilisation Distribution Overlap Index (UDOI) at 95% and 50% levels, plotted against varying mechanisms for use of private and public information

**Fig. 4:**
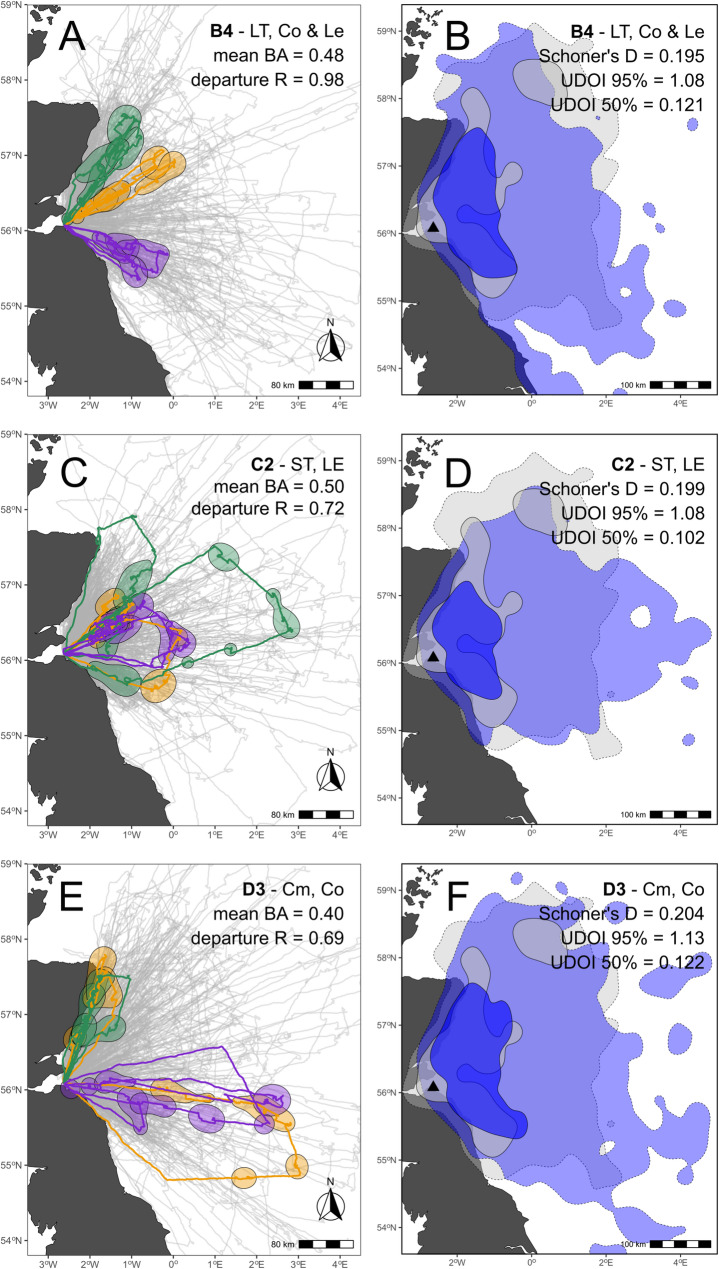
Maps of simulated gannet tracking data at the individual-level (left) and utilisation distributions (UDs) of foraging locations at the population level (right) for three trip analysis. Each row represents different simulation conditions: A & B – B4, C & D – C2, E & F – D3. Shorthand for different mechanisms implemented in those simulations: LT = long-term memory; Co = competition from conspecifics; LE = local enhancement; ST = short term memory; Cm = combined memory. In tracking data plots in left column (**A**, **C** & **E**) grey paths show all 300 trips from 100 individuals, where purple, orange, and green paths show three consecutive foraging trips from three different individuals, with differences in shades indicating different trips, and kernels of the same colour displaying the 50% utilisation distributions (UDs) of each trip based on foraging locations only. Blue contours on the UD plots in the left column (**B**, **D** & **F**) are based on locations where simulated gannets were in ARS movement displaying 95% (lighter blue and dashed border) and 50% (darker blue and solid border) UDs overlaid on grey contours showing the same respective dimensions that represent the distribution of prey in the model landscape

Memory mechanisms were the primary driver of realism in BA foraging area overlap (Fig. 3A) for three consecutive trips. All simulations including it (B1-D4) exhibited significant overlap in foraging areas compared to random assignment of bird ID to trips (Table S7), whereas models with absence of memory mechanisms (A1-A4) did not. Simulation B4 (long-term memory with enhancement and competition) was the top-ranked model by Mean Absolute Error (MAE = 0.03, Figs. 3B and 4A) with a BA mean of 0.48, compared to the observed value of 0.47 (n individuals = 98), noting that observed BA scores exhibited variation across years (Table 2). B4’s BA distribution was also closely matched to the observed equivalent (KS test: D = 0.110, *p* = 0.529, Fig. 3B, D). The short-term memory models (C) consistently aligned with observed BA levels, with all four being ranked in the top five by MAE behind B4 (Fig. 3A, B, Table S7).

Across all memory types, the inclusion of competition avoidance (3) or the combination of enhancement and competition (4) resulted in lower mean BA values compared to no social interaction (1) or local enhancement only (2) (Fig. 3A). Despite this, the top seven ranked simulations (B4, C3, C4, C2, C1, B3 & D3) all successfully replicated the underlying empirical BA distribution (Figs. 3B, D and 4, Table S7).

Empirical tracks showed moderate repeatability in departure angles (*R* = 0.54 ± 0.06, Fig. 3C; Table 2). Models without memory failed to produce any directional consistency, whereas those with long-term memory, including the BA top-ranked B4, substantially overestimated consistency (*R* = 0.98–0.99, Fig. 3C). This overestimation is an artefact of the fixed departure angle coding for mechanism B and highlights a structural mismatch. In contrast, simulations with short term memory (C) and the combined complex memory (D) provided a closer match with repeatability values (*R* = 0.69–0.84) but are still somewhat higher than the observed level (Fig. 3C), with simulation D3 (combined complex memory and competition) coming the closest. No simulations displayed repeatability of trip durations (Table S7), in keeping with empirical observations (*R* = 0 ± 0.03).

Patterns across simulations were consistent for both daylight trip duration and maximum distance from the colony, with differences being influenced more heavily by public information that private information (Fig. 3E, F). Models including competition (3 & 4) consistently showed longer daylight trip durations and greater maximum distances compared to no social interaction or local enhancement counterparts, bringing them more in line with observed values (Fig. 3E, F). Max distance and trip duration generally saw reductions with the inclusion of local enhancement (2 & 4) compared to no social interaction or solely competition (1 & 2, Fig. 3E, F, Table S7). The simulation with shortest mean daylight duration (hrs) and maximum distance (km) was B2, followed by B1 and D2 (B2: duration = 12.39 ± 5.11, max distance = 151.88 ± 91.71, B1: duration = 12.4 ± 4.97, max distance = 161.61 ± 91.37 & D2: duration = 12.53 ± 5.18, max distance = 155.23 ± 93.1).

General spatial overlap (Schoener’s D) peaked at 0.204 for simulation D3 which pairs combination memory with social competition (Fig. 3G). A similar overall pattern across mechanisms was observed for 95% UDOI, where B2 performed best (1.16; Fig. 3G, H). Overlap of core foraging areas with prey (50% UDOI) was highest in D3 (0.122, Fig. 4F), followed by D4 and B4 (0.121), with all memory types (B, C, D) generally performing better when paired with social competition and local enhancement. Regarding the distribution of departure angles most trips were not significantly different and the bimodal pattern wherein individuals are more likely to venture northeast and southeast of the colony seen in empirical data, tended to be better represented by more complex simulations (Figure S18).

**Fig. 5:**
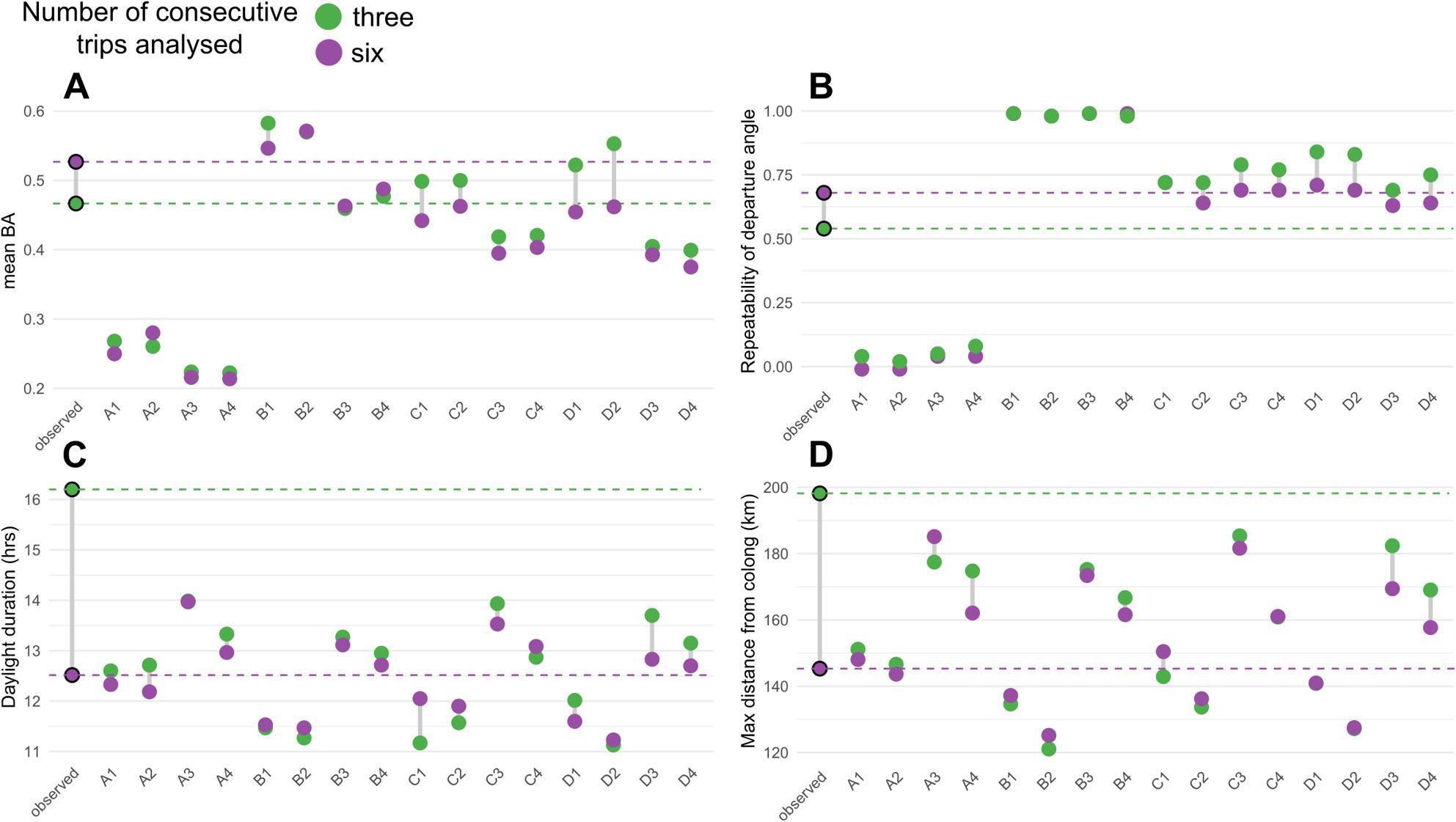
Difference between number of consecutive trips analysed (three = green points, six = purple points) for estimates of (**A**) mean Bhattacharyya’s Affinity (BA), (**B**) repeatability of departure angle, (**C**) Daylight trip duration (hrs), and (**D**) maximum distance from the colony (km) for simulations A1-D4. Observed value is shown on far left of each plot with point outlined in black

### Comparison of six and three consecutive trips

Our analysis of the subset of individuals with six consecutive trips (*n* = 33) revealed a marked increase in observed IFSF-related metrics compared to the three trip dataset (*n* = 98, Fig. 5A & B, Table 2). Observed mean BA increased from 0.47 to 0.53 (Fig. 5A; Table 2) while repeatability of departure angle rose from 0.54 to 0.68 (Fig. 5B; Table 1). In contrast, the majority of simulations exhibited a decrease in mean BA when analysing six trips, with the most pronounced declines occurring in simulations C1, C2, D1 & D2 (Fig. 5A, Table S8). However, simulations incorporating competition (3 & 4) for six trips maintained BA levels similar to three trip estimates across all memory mechanisms. For six trips, the top ranked model for BA according to MAE is B1 (mean BA = 0.55, MAE = 0.05, Figure S17, Table S8). Repeatability of departure angle dropped with more trips for the majority of simulations, aside from those with long-term memory, again, due to fixed departure angles (B1-B4, Fig. 5B).

For the subset of more consecutive trips, observed daylight trip durations and maximum distance from the colony decreased substantially (Table 2). Consequently, simulated daylight durations aligned more closely with observed data (Fig. 5C), with a similar, though less pronounced, result for maximum displacement (Fig. 5D). The number of consecutive trips analysed did not substantially alter general patterns of simulated foraging area spatial overlap with prey distributions across mechanisms (Figs. 3 & S17, Tables S7 & S8).

## Discussion

Our results demonstrate that IFSF in gannets is an emergent property of private information use, with fine-scale realism moderated by social information and memory persistence. Across all simulations, navigational memory was the fundamental driver of realistic foraging area overlap, consistent with previous work linking memory to spatial segregation in central place foragers [4, 39, 58–60]. However, the incorporation of social processes tended to enhance overlap with prey, particularly in core areas. Avoidance of competition in particular was necessary to reproduce extended trip durations and displacements seen in the empirical data, while facilitating persistence in individual foraging area overlap over more consecutive trips.

By testing mechanisms ranging in complexity against multiple hierarchical metrics across two temporal windows of trip aggregation, we aimed to mitigate equifinality [33, 61] by providing contrasting results and grounds for falsifying less-supported mechanisms. While no single simulation reproduced all observed patterns perfectly, our findings show that the integration of both private and public information is essential to approximating the observed spatial fingerprint of gannets, supporting suggestions that seabirds employ memory to locate broad-scale areas, while social information is used to fine-tune resource localisation within those localities [15].

### Memory as the primary driver of IFSF

Simulations employing memory mechanisms consistently ranked the highest for producing observed levels of foraging area overlap. In particular, the long-term memory mechanism, which imposed a fixed departure bearing, performed best when overlap was assessed across both three and six consecutive trips. This result supports findings from a similar model of Egyptian fruit bats *Rousettus aegyptiacus* where realistic spatial partitioning did not emerge from food availability and travel constraints alone but instead required memory-based decision making [59]. Similarly, intra-colony segregation in lesser kestrels *Falco naumanni* can arise from personal information and memory without social information use [60], reinforcing the idea that memory alone can structure space use at the population-level.

Despite its strong performance for foraging area overlap, the long-term memory mechanism substantially overestimated the repeatability of departure angles, indicating a structural mismatch with empirical gannet behaviour. Memory mechanisms with more flexibility in departure angle choice better approximated observed directional variability, although they still tended to overestimate departure consistency. This suggests that while gannets exhibit repeatable broad-scale overlap in foraging localities, the route selection process is likely more stochastic than how it's represented in our models.

Such overestimation may arise because departure angle choice is influenced by short-term environmental cues which we have not included, such as colony exit dynamics or wind conditions [45, 62]. Additionally, gannets may be relying less on fixed bearings and more on targeted navigation with a cognitive map towards persistent oceanographic features signalling enhanced prey abundance, such as tidal mixing fronts [4] or high fishing activity [63]. Foraging specialisation may further contribute to this pattern, where individual gannets show consistent responses to varying environmental conditions including sea surface temperature, chlorophyll-a concentration and copepod biomass [21]. Further evidence comes from Votier et al. [64] who show dietary specialisation through analysing stable isotopes, a method that Wakefield et al. [4] also used to confirm long-term specialisation of individuals over subsequent breeding seasons. Together, these findings suggest that while we may have broadly captured some decision rules, real gannets likely show flexibility between strategies in response to varying environmental conditions and are likely to target particular localities consistently.

### The role of public information

Incorporating public information altered foraging area overlap, efficiency and population-level spatial structure. While local enhancement resulted in modest increases in foraging efficiency (inferred from trip durations and displacement), competition-based avoidance reduced it. Simulations including the latter produced a greater persistence of foraging area overlap across consecutive trips. This suggests a link between competition of this kind and spatial partition, consistent with theoretical predictions that individual consistency and specialisation act to reduce intra-specific competition [1].

Our results align with Morinay et al. [60], who showed that spatial segregation intensified when individuals had good memory and experienced competitive pressures, rather than relying on social information alone. We observed a potential trade-off between efficient individual foraging and broad-scale spatial dispersal where mechanisms that promoted population-level spread tended to reduce trip efficiency. This pattern may reflect the decisions made during chick-rearing when high constraints favour access to reliable foraging areas, even when further away, over the risk of more opportunistic foraging in competitive areas closer to the colony. This interpretation is supported by empirical work showing adult breeding gannets had stronger IFSF than failed breeders [52], likely a consequence of higher constraints reducing exploratory travel, and modelling which indicated stronger spatial segregation between neighbouring colonies caused reductions in individual intake rate [40]. Therefore, our finding suggest that competition-mediated spatial structure may stabilise access to resources over time, even if it reduces short-term efficiency.

In species such as the Cape gannet which exploit ephemeral, high-density prey [65], short-lived feeding opportunities for aggregations of gannets may arise wherein intraspecific competition is temporarily reduced [26]. In contrast, observations of gannets in the North Sea indicate relatively low conspecific densities at foraging locations [27], suggesting that an avoidance mechanism may be more relevant in this system. Previous work on spatially implicit models has shown that colony segregation can emerge from memory and local enhancement, without explicit competition [39, 40]. Within colonies, we have shown that incorporating explicit conspecific detection and avoidance has increased behavioural realism, indicating that social sensing in this fashion likely contributes to gannet movement decisions.

### Consecutive trips and individual consistency

Analysing six consecutive trips generally improved correspondence between simulated and observed IFSF metrics, but these results should be interpreted cautiously due to potential sampling bias. Device retrieval had to occur within short windows, and therefore individuals with more trips recorded during this period were likely formed of shorter, potentially more consistent trips. Some observed individuals exhibited striking consistency, exemplified by one bird which travelled to the same foraging location in ten consecutive trips (Figure S21). This likely contributed the high BA scores seen in data from 2011 (Table 2), particularly for the subset for six consecutive trips where this individual would have more influence. Reaching this location required navigating around a section of land as it was not directly in line of sight from the colony, reinforcing our interpretation that individuals target particular foraging locations as opposed to simply maintaining consistent departure angles.

Despite such evidence of consistent space use, few studies have explicitly linked IFSF to foraging efficiency, as most analyses have focused on comparing mean site fidelity within and among years and/or individuals. Where links have been explored, higher IFSF has been associated with reduced foraging effort, such as in black-faced cormorants *Phalacrocorax fuscescens* where increased IFSF corresponded to lower proportions of time spent foraging per trip and reduced dive rates [7]. Similarly, Regan et al. [66] reported higher post-trip site fidelity when total foraging time was lower in common guillemots *Uria aalge* and razorbills *Alca torda*. Comparable trip-level fidelity was also found when foraging was concentrated into fewer, longer bouts, suggesting prolonged exploitation of higher value patches. Building on these findings, further decomposition of the reciprocal links between foraging effort and IFSF may help detect a spectrum of foraging modes, potentially ranging from consistent to more exploratory, and the conditions that may trigger transitions between them.

### Limitations and future directions

Despite managing to broadly capture empirical patterns, our model simplifies several aspects of gannet foraging ecology. Our movement model could be made more realistic by incorporating correlated random walks, which introduce directional persistence and produce broader-scale curvature in foraging trajectories rather than the emergence of relatively linear travel between foraging sites seen here. Such models could also include dynamic attraction to key locations [e.g. 41], such as a foraging site or the colony. This could allow movement decisions to vary with motivation across the duration of a foraging trip and remove the need to delineate inbound travel, thus permitting foraging throughout the whole duration of a trip.

As discussed above, departure angle stochasticity was limited, and a mechanism for targeting particular sites at sea was not included. The latter could be aided by representing environmental cues like tidal mixing fronts. Additionally, modelling of dynamic, rather than static, prey fields could further increase stochasticity in model outputs to be more comparable with observed levels. While the information centre-hypothesis (ICH) [22] has been shown to influence foraging directions in some gannet species [30], evidence for consistent colony-based directional cueing in northern gannets is mixed, with individuals often dispersing independently and social interactions appearing to occur primarily after departure [27, 62, 67]. We therefore prioritised local enhancement as a parsimonious representation of social information use but acknowledge that explicitly incorporating ICH or colony-level cues, alongside dynamic prey fields, as in Boyd et al. [36], and their depletion [11] over time represents an important topic for further investigation.

It takes gannets five years to reach breeding age, during which they exhibit much more exploratory trips than breeding adults [52]. When breeding they refine knowledge during pre-laying and incubation [68], a pattern reflected in lesser kestrels that show higher IFSF during nestling rearing compared to incubation [69] likely reflecting higher energy constraints. Hence, our temporal scope of learning (10 trips) was a considerable simplification. This was due to computational limitations with justification that this trade-off would offset the chance of all birds learning to forage in the same place due to our static prey map and hence be more representative of the stochasticity in fidelity to particular foraging sites among individuals in reality. Longer term GPS deployments on gannets across the whole breeding season, which improved technology now permits, would allow these dynamics to be tested explicitly. Furthermore, acquiring such an expansive dataset would permit investigation into how the number of consecutive trips analysed influences interpretation, and the possibility that different groups of birds employ different information strategies; a potential alternative explanation for the considerably higher observed BA estimates for the subset of six consecutive trips. More sophisticated memory coefficients, following the approach of Lourie et al. [59], could also be incorporated if the methods can be adapted to a marine context, where the absence of fixed landscape features makes quantifying IFSF more challenging than in terrestrial systems. One possibility could be the use of step-selection analyses that incorporate memory covariates [70], in which multiple time-window occurrence distributions could be tested to assess the weighting of short- and long-term spatial memory.

Social information was implemented heuristically, so improving its parameterisation to strengthen inference would be a priority for future empirical research. Although a challenging endeavour, it is possible at small colonies by simultaneous tracking of the majority of individuals [30]. This presents issues of scale if looking to extrapolate to larger colonies, such as Bass Rock, but other possibilities such as radar [71], animal-borne cameras [31, 67] or other remote sensing techniques could assist in characterising the processes shaping these patterns.

## Conclusions

Our findings advance understanding of colonial seabird foraging by demonstrating, through a spatially explicit and mechanistic modelling approach, that IFSF in gannets emerges from a hierarchical decision-making process. Long-term memory governs broad-scale space use, while short-term social cues refine movement at finer scales. The variable performance of different mechanisms across scales suggests that no single information source dominates across contexts. Instead, gannets likely shift flexibly between memory- and socially mediated strategies, producing a spatial organisation that is stable yet adaptable. In an era of rapid environmental change [72], mechanistic movement models such as this are essential for prediction and conservation [73]. Our approach provides a robust framework for advancing ecological theory and identifies clear empirical targets for testing how colonial species balance private and public information when navigating complex environments.

## Electronic Supplementary Material

Below is the link to the electronic supplementary material.

Supplementary Material 1

## Data Availability

Gannet tracking data used in this study are available to request from the BirdLife International Seabird Tracking Database (https://www.seabirdtracking.org/) with the following dataset IDs: 718, 955-956, 1341-1342, 1472. The NetLogo code for movement simulations, and associated files required to run are available on GitHub (https://github.com/ChrPol/gannet_movement_IBM).

## Abbreviations

IFSF: Individual foraging site fidelity
IBM: Individual-based model
POM: Pattern-oriented modelling
UD: Utilisation distribution
BA: Bhattacharyya’s affinity
