# Supplementary material for: Movement simulations reveal both memory and social information drive individual foraging site fidelity in gannets
Source: Mov Ecol. 2026 May 14;14:50. doi: 10.1186/s40462-026-00656-8 (PMC13343878; doi:10.1186/s40462-026-00656-8)
Supplement: Supplementary file 1 — Supplementary Material 1 [file 40462_2026_656_MOESM1_ESM.docx]

Supplementary material - Movement simulations reveal both memory and social information drive foraging site fidelity in gannets

Contents

[1 Introduction 2](#_Toc226888641)

[2 Biological background 3](#_Toc226888642)

[2.1 Study site and foraging trips 3](#_Toc226888643)

[2.2 Foraging site fidelity 5](#_Toc226888644)

[3 Empirical data and patterns 8](#_Toc226888645)

[3.1 Telemetry data collection 8](#_Toc226888646)

[3.2 Patterns extracted: movement model 8](#_Toc226888647)

[3.3 Patterns extracted: IFSF simulation experiments 9](#_Toc226888648)

[3.4 Prey density grid 12](#_Toc226888649)

[4 Model description following ODD protocol 13](#_Toc226888650)

[4.1 Purpose 13](#_Toc226888651)

[4.2 Entities, state variables, and scales 13](#_Toc226888652)

[4.3 Process overview and scheduling 16](#_Toc226888653)

[4.5 Design concepts 18](#_Toc226888654)

[4.5.1 Basic principles 18](#_Toc226888655)

[4.5.2 Emergence 19](#_Toc226888656)

[4.5.3 Learning 19](#_Toc226888657)

[4.5.4 Sensing 19](#_Toc226888658)

[4.5.5 Interaction 19](#_Toc226888659)

[4.5.6 Stochasticity 20](#_Toc226888660)

[4.5.7 Observation 20](#_Toc226888661)

[4.6 Initialisation 20](#_Toc226888662)

[4.7 Input data 21](#_Toc226888663)

[4.8 Submodels 21](#_Toc226888664)

[5 Modelling conditions for simulation experiments 26](#_Toc226888665)

[5.1 Private information 26](#_Toc226888666)

[5.1.1 Long-term memory 26](#_Toc226888667)

[5.1.2 Short-term memory 26](#_Toc226888668)

[5.1.3 Combined memory 27](#_Toc226888669)

[5.2 Public information 27](#_Toc226888670)

[5.2.1 Local enhancement 27](#_Toc226888671)

[5.2.2 Competition 28](#_Toc226888672)

[5.2.3 Combination of local enhancement and competition 28](#_Toc226888673)

[6 Parameterisation 28](#_Toc226888674)

[6.2 Movement model 28](#_Toc226888675)

[6.2.1 Methods 28](#_Toc226888676)

[6.2.2 Results 30](#_Toc226888677)

[6.3 IFSF simulation experiments 36](#_Toc226888678)

[6.3.1 Methods 36](#_Toc226888679)

[6.3.2 Results 38](#_Toc226888680)

[7 Sensitivity analysis 39](#_Toc226888681)

[7.1 Methods 39](#_Toc226888682)

[7.2 Results 39](#_Toc226888683)

[8 Evaluation 40](#_Toc226888684)

[8.1 Movement model 40](#_Toc226888685)

[8.1.2 Methods 40](#_Toc226888686)

[8.1.3 Results 40](#_Toc226888687)

[8.2 IFSF simulation experiments results 42](#_Toc226888688)

[9 Empirical support for modelling decisions 50](#_Toc226888689)

[References 51](#_Toc226888690)

# 1 Introduction

In this document we describe in more detail the methods used for modelling simulations of chick-rearing Northern gannets (*Morus bassanus*, hereafter “gannet”) at Bass Rock, beginning with a description of the biological background of (i) the study site and foraging trips to be used in movement simulations, and (ii) the potential mechanisms which may be involved in driving individual foraging site fidelity (IFSF) patterns. We then describe the telemetry data collection, processing and usage for this study, followed by the various patterns construed from this data and relevant literature, which were employed in a pattern-oriented modelling (POM, (Grimm & Railsback, 2012)) approach to guide development, parameterisation and evaluation of our model.

We then provide a full description of our model using the Overview, Design Concept, Details (ODD) protocol (Grimm et al., 2006, 2020), which is a format for the standardised communication of individual-based models. Following the ODD, we describe how different simulation experiment hypotheses were modelled and included more detailed descriptions of the development and testing of the model with an expanded results section.

# 2 Biological background

## 2.1 Study site and foraging trips

The breeding colony at Bass Rock (56° 4’ N, 2° 38’ W) had 75,259 apparently occupied sites (AOS) in June 2014 (Murray et al., 2015) making it the world’s largest gannet colony. However, the population has seen substantial declines following a severe outbreak of highly pathogenic avian influenza (HPAI) in 2022 (Lane et al., 2023). As a consequence of this population density, they experience relatively high levels of intraspecific competition during the breeding season when acting as central place foragers, resulting in the longest foraging trip durations and distances travelled when compared with other colonies in the UK and Ireland (Lewis et al., 2001; Wakefield et al., 2013).

Figure S1 depicts a foraging trip that was 16.1 hours long, where a distance of 638 km was travelled, leading to a maximal distance of 267 km from the colony. This particular trip began at dawn with the gannet returning to the colony at 20:56 h. Outbound travel is relatively straight, with occasional ARS zones, before the trip trajectory begins to arc around where ARS zones are most highly concentrated around the outer arc of the trip. Inbound travel is defined as the journey back towards the colony on completion of the arc (~180° turn in orientation), which is characterised by similar linearity as the outbound trip (Pettex et al., 2010), with little to no ARS behaviour taking place. From this we deduce that the individual in question has made the decision to stop foraging and head back to the colony, which is likely due to the required amount of food being acquired for sustenance of themselves and their chick.


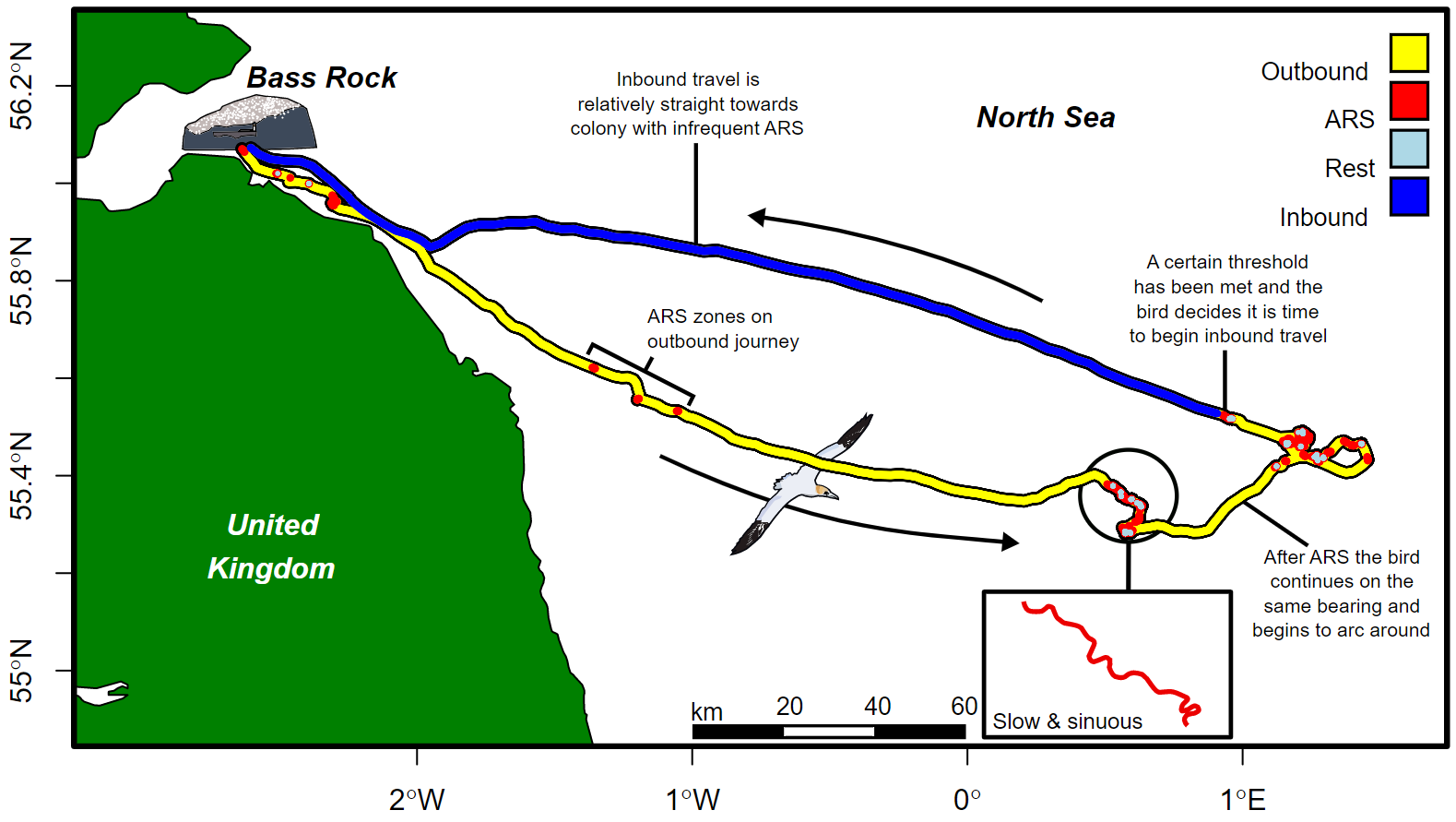


Figure S1: Annotated single foraging trip from a chick-rearing Northern gannet breeding on Bass Rock in 2015.

Commuting to and from the colony is characterised by relatively high speeds and a straight direction of travel. In contrast, when birds enter ARS movement they slow down considerably and the path becomes much more sinuous (Figure S1), likely allowing for greater inspection of the sea surface in the local area in order to visually locate and identify prey. Gannets dive to obtain a wide range of prey species (Hamer et al., 2000), but their success rate and as such their functional response (relationship of prey obtained to prey density) is not well known. However, it has been shown that most dives take place within ARS movement, and two thirds of dives take place on the outwards phase of the trip, i.e. before reaching the maximal distance from the colony (Hamer et al., 2009).

ARS bouts are punctuated with periods of resting on the sea surface, and a single trip will typically have many short rests of < 20 mins, and one or two longer periods of rest. In this trip (Figure S1) there were 22 rests that were < 20 mins, with 3 slightly longer periods of rest (26, 32 & 34 mins). Inspection of other empirical trips shows that it is not uncommon for these daytime rests to last over an hour. Gannets typically have short rests after each dive bout, and some authors have hypothesised that foraging at the beginning of the trip allows birds to feed themselves, followed by a period of resting and digesting prey before commencing foraging for food for the chick (Ropert-Coudert et al., 2004).

In this foraging trip (Figure S1) over half (58%, 9.27 hours) of the time was spent travelling, with 72% of this being outbound travel. Just less than a quarter of the trip (21%) was spent in ARS and resting respectively. This trip spans the duration of daylight hours, but often trips can be over 24 hours in length where gannets spend the night at sea. As gannets are visual predators they do not forage at night, and mostly rest on the sea surface (Furness et al., 2018). Consequently, these trips will tend to have a higher proportion of resting time.

One parent remains with the chick while the other is on a foraging trip (Nelson, 2010). On return, it is typical to see a brief overlap where both parents are at the nest before the other parent departs on a foraging trip. Consequently, the time spent by an individual at the colony between trips will be very similar to the duration of the foraging trips at that colony. The diet of gannets at Bass Rock consists of a wide range prey species of different sizes (Hamer et al., 2000) including sandeels (*Ammodytes spp.*), mackerel (*Scomber scombrus*), herring (*Clupea harengus*) and sprat (*Sprattus sprattus*). Information available on the weight of prey items is scarce but Garthe et al. (Garthe et al., 1999) reported a maximum food quantity of 745 g with 101 g being the median.

## 2.2 Foraging site fidelity

Gannets at Bass Rock show foraging site fidelity within and across years (Wakefield et al., 2015) and individuals depart the colony in a consistent direction, which is a pattern also known to exist at other colonies (Patrick et al., 2014; Soanes, Atkinson, et al., 2013). From this consistency it’s possible to infer that there may be a degree of reliability in the broad scale distribution of prey over long time periods, or it may be that site familiarity, learned through earlier life, leads to a bet-hedging strategy of visiting the same area on successive trips (Grecian et al., 2018). However, gannets do not show complete fidelity to foraging sites throughout the chick-rearing period, and in Figure S2 individual 1484906 has markedly different departure bearings and little overlap between foraging locations during three consecutive trips. This pattern has been observed at this population consistently (Hamer et al., 2001; Wakefield et al., 2015) and could indicate that individuals decide to forage in alternative locations, which may be the result of poor success in that particular area on a previous trip.

Further to the use of private information, there is also a wealth of public information available to gannets especially at large colonies (Tremblay et al., 2014) which may provide cues on where, and perhaps where not to find prey. The use of public information at the colony to indicate the direction of successful foraging from returning conspecifics, termed the “information-centre” hypothesis (Ward & Zahavi, 1973) has been shown to influence movement patterns and foraging locations in a small population of close relatives (*Morus serrator*), where birds frequently associated with conspecifics while departing from the colony, resulting in co-departing birds having similar initial foraging patches (Jones et al., 2018). However, Northern gannets often join rafts offshore before departing on foraging trips, probably for purposes such as preening feathers after being at the colony, with no indication that joining such rafts influences foraging trip distance or duration (Carter et al., 2016). Moreover, it is unlikely that individuals would show consistency in departure direction if they were subject to cues from the colony, and gannets have been observed to disperse from Bass Rock individually (Camphuysen, 2011). Therefore, it is likely that most social interaction is done after departing the colony.

In Figure S2, individual 1459919 (yellow tracks) represents the average level of fidelity observed in the empirical data. There is some overlap in foraging locations, but there are new locations in each trip and there is slight variation in departure angle from the colony. We hypothesise that this is the typical pattern because gannets recall broad scale areas to forage in and depart from the colony at a roughly consistent angle, and will typically travel in a linear fashion towards this area but can deviate from this bearing if they obtain cues from conspecifics to where suitable foraging patches are located, and switch course towards such potential foraging opportunities (Thiebault et al., 2014).


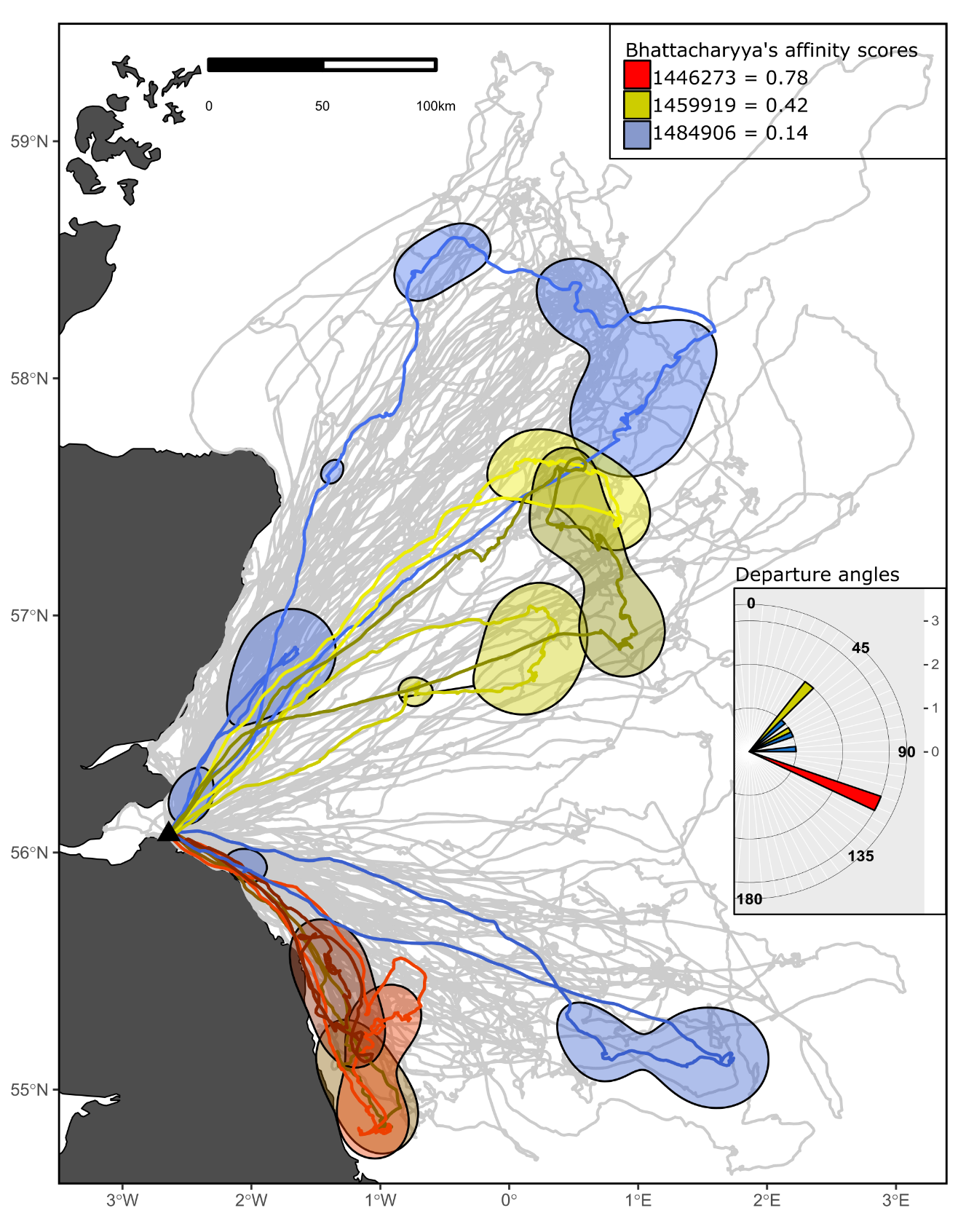


Figure S2: Map of empirical gannet tracking data in the North Sea to display the patterns associated with individual foraging site fidelity (IFSF) from the colony at Bass Rock (black triangle). All data are from the chick-rearing period in 2015. Grey lines show 114 trips from 29 individuals. Blue, yellow, and red lines show three foraging trips from three different individuals, with kernels of the same colour displaying the 50% utilisation distributions (UDs) based on foraging locations only. Bhattacharyya’s affinity (BA) scores provide quantification of the mean overlap of foraging distributions within each individual by taking a mean of the three pairwise combinations (Note: the UDs are pictured are not those used to quantify BA but instead are included as visual representation). The histogram inset on the right shows the departure bearings (10km from the colony).

# 3 Empirical data and patterns

Empirical data were used for two main purposes; (i) to extract key patterns of individual foraging trips and those relating to IFSF to guide model development, parameterisation and evaluation, and (ii) to assign approximate prey density to cells of the landscape in the model from where birds foraged in reality.

## 3.1 Telemetry data collection

Movement data from chick-rearing adults were obtained in 2011, 2012, 2015 and 2016 using GPS loggers (igotU-GT600, Mobile Action Technology, Taipei, Taiwan) attached to the upper side of the central tail feathers with tape and set to record locations at 2 min intervals (Lane et al., 2019; Wakefield et al., 2015). The whole dataset comprised 504 foraging trips from 118 individuals, which means it is highly likely that this has captured the home range of the Bass Rock gannet colony (Soanes, Arnould, et al., 2013).

## 3.2 Patterns extracted: movement model

Table S1 below displays the different patterns that were extracted from empirical data and reported values (if applicable). To calibrate fine scale movement in the model, GPS data were interpolated to 2 min intervals to extract turning angles and step lengths for behaviour assigned to commuting, ARS and rest. For broad scale movements a linearity index was calculated for each foraging trip where a value closer to 1 would indicate a more linear trip:

Linearity index for foraging trip *i* = Total length of trip *i* / (Maximal distance of trip *i* * 2)

Another simple metric (difference in bearing) was extracted to approximate the arc of a trip and where:

Difference in bearing for trip *i* = abs (Bearing at distal point of trip *i* – initial bearing of trip *i*)

where the distal point of the trip is the location furthest from the colony and the initial bearing is at a distance of 10 km from the colony on the outbound portion of the trip (hence excluding initial orientation and activities such as bathing before commencing on directed travel). Other commonly used trip metrics including duration (h), length (km) and activity budgets (proportion of time spent in different behaviours) were extracted from the empirical data to be used in parameterisation and evaluation, as described in Table S1 below.

Table S1: Summary statistics of key patterns extracted from empirical data used in the pattern-oriented modelling process to guide development and evaluation of the movement IBM.

| Pattern | Classification | Where in the modelling process was it used? | Empirical values (if applicable) |
| --- | --- | --- | --- |
| Activity budgets | Behaviour | Validation and analysis | Some interannual variation, but typically 37%: 37%: 26 %, travel: forage: rest per trip. Typically, less travel at smaller colonies (Wakefield et al., 2013) |
| Trip duration | Movement | Parameterisation – functional response | 19.2 ± 8.8 hours |
| Trip length | Movement | Evaluation | 386.4 ± 226.6 km |
| Linearity | Movement | Parameterisation – orientation | 1.49 ± 0.36 |
| Bearing difference | Movement | Parameterisation – orientation | 14.41 ± 14.64 ° |
| Step lengths | Movement | Calibration | Travel = 1783 ± 371 m/2min  ARS = 862 ± 527 m/2min |
| Turning angles | Movement | Calibration | Travel sd = 0.164  ARS sd = 0.949 |

## 3.3 Patterns extracted: IFSF simulation experiments

To evaluate how well the models predicted the key features associated with IFSF in the real population, we compared patterns extracted from the model outputs with those observed in empirical data (Table S2). The latter comprised GPS tracking data of three consecutive trips from 98 individuals (*n* individuals 3 trips: 2011 = 21; 2012 = 30; 2015 = 25; 2016 = 22; total trips = 294), and a subset of six consecutive trips from 33 individuals (*n* individuals 6 trips: 2011 = 13; 2012 = 9; 2015 = 6; 2016 = 5; total trips = 198), tagged during the chick-rearing period at Bass Rock in July and August during those years (Lane et al., 2019; Wakefield et al., 2015). Data from the models were “collected” in a similar fashion, i.e. as coordinates observed in two-minute timesteps, following the “virtual ecologist” approach (Zurell et al., 2010), with a separate analyses of three consecutive trips and six consecutive trips per individual to compare to the observed data.

Table S2: Patterns used in calibration and evaluation of the model for the IFSF simulation experiments

| Phase of modelling cycle | Pattern | Hierarchical level | Category | Empirical value (if applicable) |
| --- | --- | --- | --- | --- |
| Development/calibration | Visual comparison of tracks | Individual and population | Movement | - |
|  | Activity budget of foraging trips | Individual and population | Behaviour | 37%: 37%: 26 %, travel: forage: rest per trip |
| Evaluation - IFSF | Repeatability of departure angle (i) | Individual | Movement | 3 trips = 0.54 ± 0.06 |
|  |  |  |  | 6 trips = 0.68 ± 0.07 |
|  | Repeatability of trip duration (ii) | Individual | Movement | 3 trips = 0 ± 0.03 |
|  |  |  |  | 6 trips = 0.11 ± 0.06 |
|  | Individual consistency in use of foraging areas using Bhattacharyya’s affinity (BA) score – mean (min, max) (iii) | Individual | Spatial | 3 trips = 0.47 (0.12, 0.99) |
|  |  |  |  | 6 trips = 0.53 (0.26, 0.99) |
| Evaluation – foraging  efficiency and population patterns | Similarity of the simulation’s foraging utilisation distribution to the empirical distribution used to inform prey landscape using UDOI (iv) | Population | Spatial | 3 trips  95% = 1.298, 50% = 0.219 |
|  |  |  |  | 6 trips  95% = 1.213, 50% = 0.195 |
|  | Average daylight trip duration - hours (v) | Population | Behaviour | 3 trips = 16.4 ± 8.3 |
|  |  |  |  | 6 trips = 13.8 ± 7.1 |
|  | Average furthest distance from the colony - km (vi) | Population | Movement | 3 trips = 198 ± 103 |
|  |  |  |  | 6 trips = 162 ± 97 |
|  | Distribution of departure angles (vii) | Population | Spatial | See Figure S18 |

For both model outputs and empirical data, standard trip metrics were extracted (Table S6) including duration (h), length (km), furthest distance from the colony (km) and a linearity measure. To evaluate how well each model reproduced IFSF three patterns were quantified as follows:

1. *Repeatability of departure angle* – we report the repeatability (R) of the departure angle of three consecutive trips of 30 individuals, computed using circular ANOVAs, for which R values were obtained following (Lessells & Boag, 1987) with associated standard errors from (Becker & Others, 1975). Higher values indicate that within-individual variance is lower than between-group variance.
2. *Repeatability of trip duration* – The repeatability value for trip duration and its standard error was reported along with a p-value testing the null hypothesis that within-individual variation is equal to between-group variation. This was achieved using the R package ‘rptR’ and a GLMM structure (Stoffel et al., 2017).
3. *Bhattacharyya’s affinity (BA)* – This measure of individual consistency in the use of foraging areas was calculated following (Wakefield et al., 2015). Kernel densities were estimated on each foraging trip, including only those locations where individuals were exhibiting foraging behaviour, using the package ‘adehabitatHR’ (Calenge & Calenge, 2018) using a fixed smoothing parameter (bandwith) of 15,000 m. Then the overlap of the resulting utilisation distributions (UDs) was calculated using Bhattacharyya’s affinity (Fieberg & Kochanny, 2005) of all pairwise combinations of the first three trips recorded for each individual in the population in question. A score of zero indicates that there is no overlap between UDs of a certain individual’s trips, whereas 1 indicates perfect overlap, indicating that the same foraging areas have been used on consecutive trips. To test if the IFSF of each population (model outputs or empirical data) was greater than expected by chance we created a null distribution of BA scores using a randomisation procedure, where if the null hypothesis was rejected using of a Wilcoxon ranked sum test, then we could infer that the observed IFSF differed significantly from a random assignment of bird ID to trips. To further evaluate model performance, we calculated the Mean Absolute Error (MAE) to quantify the magnitude of the difference between simulated and observed BA scores per individual. Finally, a Kolmogorov-Smirnov (KS) test was employed to compare the simulated and observed distributions of BA scores, determining whether the model successfully replicated the overall spread and shape of individual consistency observed in the empirical data.

The dataset was also evaluated for four additional patterns with the desired goal of assessing which simulation was the most efficient for individuals, which best exploited the available area of the prey distribution and which best represented population-level patterns seen in the empirical data.

1. *Utilisation distribution (UD) overlap with observed distribution* – To assess how informed the population in question was about the putative distribution of available prey in the model we compared the 95% and 50% UDs from the population in question to the equivalent UDs of the GPS data (foraging locations only) used to create the prey landscape. To quantify the space use sharing between the two UDs being compared we used the utilisation distribution overlap index (UDOI) (Fieberg & Kochanny, 2005) within the ‘adehabitatHR’ package (Calenge & Calenge, 2018). Values are typically between 0 and 1 but can exceed 1 if the two UDs being compared are nonuniformly distributed and have a high degree of overlap. Additionally, we compared simulated foraging locations directly to the underlying prey landscape using Schoener’s D index. Calculated as *D =* 1 – 0.5 ∑|*p^­^_i_ – q_i_*| where *p^­^_i_* and *q_i_* are the proportions of locations in each grid cell for the simulated tracks and prey distribution, respectively. The index ranges from zero (no overlap) to 1 (complete overlap). This dual approach was employed because, while UD-based metrics like UDOI evaluate the general spatial "footprint" and intensity of use, Schoener’s D allows for a direct, cell-by-cell comparison of resource selection against the raw environmental availability.
2. *Average daylight trip duration (h)* – This metric was selected with the perspective that the lower the daylight trip duration the more efficient that particular trip was in finding enough resources to elicit returning to the colony to feed the chick (Hamer et al., 2007).
3. *Average furthest distance from the colony (km)* – This is another measure of space use, where if gannets were foraging further afield it possibly indicates higher competition, or individuals which are less informed about their environment.
4. *Distribution of population departure bearings –* We compared the population-level distribution of departure bearings for each simulation with that of the empirical data using two-sample Kolmogorov-Smirnov tests with the null hypothesis being no difference between the simulated and empirical distribution.

## 3.4 Prey density grid

To obtain a prey density grid (Figure 1D & S3) GPS positions were interpolated to 10 second intervals using the package ‘adehabitatLT’ (Calenge & Calenge, 2018) to account for any irregularities. We then assigned the behaviour of the gannets during each trip into the category commuting, ARS or resting according to thresholds in speed and track tortuosity (Grecian et al., 2018; Wakefield et al., 2013). The ARS category was defined when GPS points had a tortuosity < 0.9 and a speed >1 m/s, indicating areas where gannets foraged. These locations were then used to calculate the time spent in a 2 x 2 km cell of a predefined grid around the colony using the ‘Trip’ package (Sumner, 2016). This grid was exported from R as a raster and imported into NetLogo (Wilensky, 1999) using the ‘GIS’ extension.

# 4 Foundational model ODD

We developed a spatially explicit individual-based model (IBM) to simulate the movements of chick-rearing gannets at Bass Rock. This model was implemented in NetLogo version 6.1.1 (Wilensky, 1999), and processing and analysis of model outputs was conducted in R (R Core Team, 2020). In the following section we describe the model according to the ODD protocol. The majority of this description is based on our foundational model with the purpose of capturing realistic movements of foraging trips, but some modifications were required to represent behaviours relating to the use of public and private information for our individual forging site fidelity (IFSF) simulation experiments. Where these arise, we indicate in bold type at the end of relevant subsections as such: “**IFSF simulation experiment modifications**”.

## 4.1 Purpose

The aim of this model was to capture the movements of chick-rearing gannets during a foraging trip from Bass Rock, both at fine- and broad- temporal and spatial scales, when foraging in a marine landscape with heterogeneously distributed prey with foraging decisions being dependent on their current environment and their previous foraging success on the current trip.

**IFSF simulation experiment modifications:** The purpose of this model is to investigate how sources of public and private information may be used across various foraging strategies, in attempt to decipher the underlying mechanisms which drive IFSF.

## 4.2 Entities, state variables, and scales

The main entity in this model was adult gannets, which are hence forth referred to as *aGannets*; a portmanteau of “agent gannet”. *aGannets* interact with their landscape, where cells representing the North Sea have a value of prey density assigned to them, and land cells are actively avoided using the avoidance submodel detailed below. One cell is defined as Bass Rock, according to its location in the Firth of Forth, and *aGannets* are aware of where this is located and when they have returned to the colony. They are characterised by their location, speed, turning angle, current movement mode, and how much food intake there has been on the current trip. The focus of this model is individual trips; thus, it is assumed that an *aGannet’s* chick is alive for the duration of the simulation, and their partner is assumed to share the duties of parental care where alternating foraging trips are made in order to meet the energy needs of the chick (Nelson, 2010). State variables are listed in Table S3 below.

Model architecture consists of a grid of 95,472 (272 x 351) square cells which each represent 4 km^2^ (2 km x 2 km) thus representing a total area of 381,888 km^2^. This area is representative of the extent of the Bass Rock colony’s chick-rearing season home range deduced from empirical data, with an extended buffer of ~50 km beyond this so that *aGannets* are not spatially constrained. It has a cell assigned for the colony, which is surrounded by sea (78,856 sea cells, 83%) representing the North Sea and extending beyond any of the recorded empirical tracks for chick-rearing adult gannets. There are 16,616 land cells (17%). Prey is distributed heterogeneously in the sea cells through each cell having an attributed prey density assigned to it when the model is being initialised. This is determined by the amount of time gannets spent foraging in this area multiplied by a constant to allow for different levels of prey density to be implicated during simulations. Towards the distal fringes of the colony’s home range, prey density increases radially with distance (Figure S3) so that *aGannets* are very likely to detect prey before reaching the edge of the landscape. On the rare occasion this does occur, it results in a decrease in that trip’s abilities to reproduce a visually realistic foraging trip but does not influence resulting trip metrics. An alternative would have been to specify a maximum range for *aGannets*, at which point they would turn back to the colony, but this may have compromised the emergence of trip trajectories as intended.


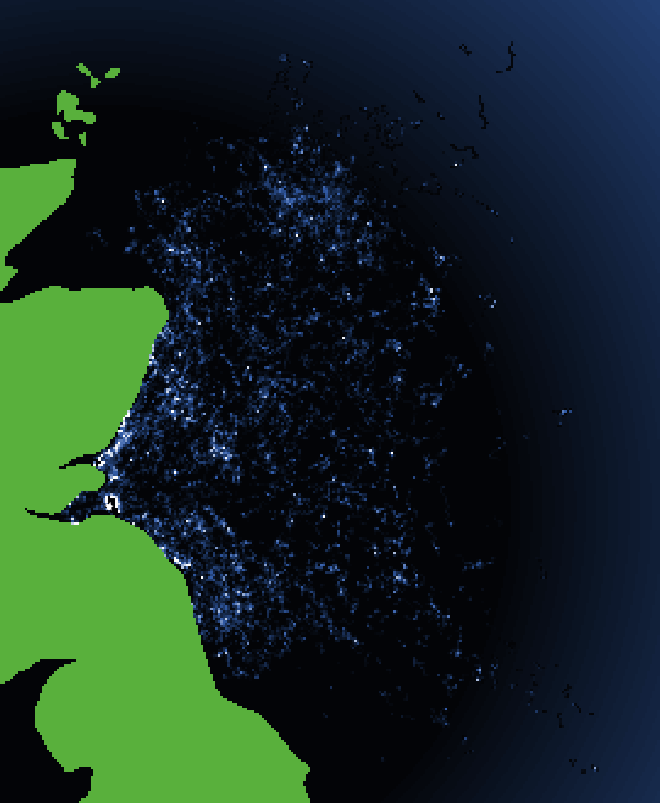


Figure S3: The model landscape as seen in the NetLogo interface, where increasing lightness in shade of blue indicates higher prey density as inferred from areas where gannets foraged according to GPS data or at the edges of the model where prey increases radially to reduce the chance of aGannets reaching the edge of the landscape.

The model runs in two-minute timesteps for a total of 91 days, representative of the length of the chick-rearing period. This timestep was chosen as this is the temporal resolution at which the empirical data were collected, thus allowing for direct comparison of model outputs with empirical patterns derived from the same data, representing the “virtual ecologist” approach (Zurell et al., 2010).

Table S3: State variables used in the movement IBM

| **State variable** | **Unit (if applicable)** | **Description** |
| --- | --- | --- |
| **Globals** | | |
| *minute* | min | What minute of the day it is |
| *day-night* | day/night | Whether it is day or night in the model currently |
| *day* | d | Which day of the simulation it is |
| **Adults** | | |
| *Food-intake* | grams | Food intake on this particular trip |
| *behav* | outbound/  ARS/ rest/ inbound/nest | Behaviour the bird is exhibiting |
| *ARS-fish-counter* | integer | How many fish has the bird ingested during current ARS bout |
| *clockwise* | Boolean | If true the trip will be a clockwise arc, and false means the bird will travel in an anticlockwise arc. |
| *Target* | XY coordinates | This is the patch that a bird recalls from assumed previous memory |
| *original-bearing* | degrees | At the beginning of travel the bearing towards the target patch is recorded |
| *head-current* | degrees | The current direction that an individual is facing in outbound travel, which is derived from the original-bearing and an adjustment based on food-intake on the current trip. |
| *ARS-chance* | arbitrary | The chance of beginning ARS when in outbound travel, which is dependent on the unknown parameters “prey-detect”, “ThresholdARS” and the prey density of the current patch. |
| *Home-bearing* | XY coordinates | The bearing leading back to the colony when the individual beings inbound travel |
| **Patches** | | |
| *Prey-density* | arbitrary | A proxy of the number of fish currently available to individuals foraging on this patch |
| *use* | - | Defines the colony patch as home, used to define whether or not the individual has reached home on its inbound travel |
| *categ* | land/water | Defines if the patch is land or water. Land patches are avoided in the avoidance procedure. |

## 4.3 Process overview and scheduling

Processes: At each time step *aGannets* are aware of their current behaviour and assess their foraging success thus far in the trip (food-intake) alongside assessing their local environment for prey in order to decide whether to remain in this same behaviour or switch to a different behaviour. Following this, behavioural counters are updated. If activated, individual output files for gannets are updated to their respective .csv files with the minute, day, trip-number, behaviour, and x and y coordinates.

In this model different processes are enacted depending on what current behavioural state the respective *aGannet* is experiencing. As such, what follows is a logical description how a typical foraging trip will proceed in the model, for which a schematic diagram can be seen in Figure S4. An *aGannet* at the beginning of the simulation will be on its nest at the colony. For the first trip there is a 0.5% chance of departing the colony at each timestep which translates to departing between 0-8 hours from the beginning of the simulation so that all *aGannets* in the simulation do not depart in synchrony. When beginning their outbound journey from the colony they pick a target patch using the “decide-heading” submodel. Orientation of the trip is decided with an equal chance of being clockwise or anticlockwise. On the outset of the outbound journey the bearing from the colony is recorded (original-bearing) to be used in subsequent orientation which is dependent on food intake.

At Bass Rock the average distance of the first dive of each trip is 54 km (Hamer et al., 2009), which was approximated in the model by having threshold of one hour (Average speed of 14 m/s translates to 50 km travelled in an hour) of outbound flying before there is the possibility of switching to ARS movement. Once an *aGannet* has gone past this threshold, it may switch movement modes to ARS through the “Enter-ARS?” submodel wherein depending on the prey density of the patch it is currently on and two unknown parameters (thresholdARS, prey-detect) which dictate the parameters of the functional response the modelled birds have to prey density (see Section 4.8 for more details), which is an approximation of type II response (Holling, 1959).

During ARS, foraging attempts may be made (submodel: “foraging-attempt”) which require visual detection of prey first, and this is based on the same type II functional response which has been approximated for the “Enter-ARS?” submodel. This is calculated at each timestep so if an *aGannet* during a particular ARS bout crosses the boundaries of one cell into another then the relevant prey density assigned to the cell currently occupied can influence predator-prey dynamics in a spatially realistic way. The parameters used here are the same as “Enter-ARS?” to avoid unnecessary complexity, and since the movement of an *aGannet* during ARS movement is slower and more sinuous, this is where it increases its chances of capturing prey in a potentially profitable area.

After capturing prey an *aGannet* will rest for either a short period or a longer period if they have reached the food intake requirement (Table S3), where rest length is drawn from a normal or gamma distribution (submodel: “decide-rest-duration”) informed by empirical data. During a particular ARS bout, if an *aGannet* has spent over 40 mins in total foraging, excluding any rests, and it hasn’t yet acquired the required amount of food it will resume outbound travel. At this point the “calculate-bearing” submodel may bias the direction of travel to begin turning back towards the colony where the relative angle to the colony is dictated by a logistic relationship with how much food has been ingested.

After successive ARS bouts, the *aGannet* will have caught the required amount of food to signal a large rest which is followed by inbound travel. This is a biased random walk towards the general direction of the colony, assuming that individuals can orientate themselves as such (Pettex et al., 2010), which is based on the distance of an *aGannet* to the colony (submodel: “find-distance-home”) and an adjustment to the previous bearing according to the current bearing of the colony from an *aGannet* (submodel: “calculate-inbound”).


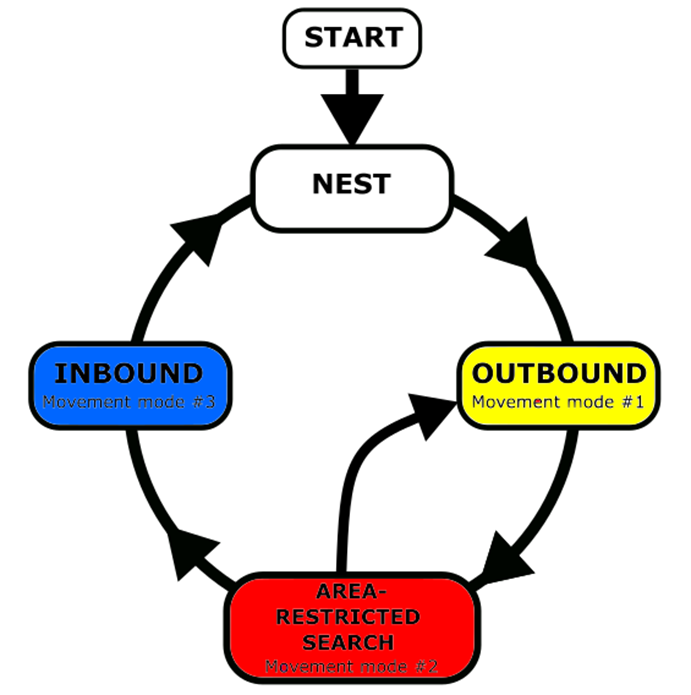


Figure S4: Schematic diagram of the different behavioural states which an aGannet may be exhibiting at any given timestep. The colours are consistent with how these movement modes are depicted in figures throughout this chapter.

Upon return to the colony behavioural counters for that trip are tallied and then reset to zero for the next trip. The duration of time to spend at the colony before departing for the next trip is drawn from a normal distribution given by the mean and standard deviation of the duration of the last 100 trips completed by any individuals. Thus, time spent at the colony will be roughly the length of trips being exhibited at any given point in the simulation. This cycle then repeats until the end of the simulation. Minute 990 during each day indicates the beginning of night, when no matter what the current behaviour or location of the individual, behaviour is set to “rest”, as it has been shown that gannets are almost completely inactive during the night (Furness et al., 2018).

Table S4: Parameter estimates used in the IBM

| **Parameter** | **Value** | **Source** |
| --- | --- | --- |
| Step length - travel | 1783 ± 371 m / 2min (~15 m/s) | Empirical data |
| Step length - ARS | 862 ± 527 m / 2min (~7 m/s) | Empirical data |
| Turning angle - ARS | 0 ± 0.949 ° | Empirical data |
| Prey size | 100 ± 25 g | (Garthe et al., 1999) |
| Food intake requirement | 900 g | Derived from digestion rate and assumption of intake of 1 fish per hour for 10 hours |
| Longer rest duration | 20 – 200 min | Empirical data |
| Shorter rest duration | 0 – 20 min | Empirical data |
| ARS bout length | Total of 40 min searching per bout | Empirical data |
| Prey detection | Parameterised value = 0.125 | Determined through parameterisation |
| ThresholdARS | Parameterised value = 20 | Determined through parameterisation |
| First ARS bout after beginning trip | 53.9 km from colony | (Hamer et al., 2009) |

**IFSF simulation experiment modifications:** During movement modes it is possible for gannets to uptake local enhancement opportunities or avoid areas that are occupied by a particular number of conspecifics.

## 4.5 Design concepts

### 4.5.1 Basic principles

This model is built on the principles of optimal foraging theory, where *aGannets* are attempting to meet their required food intake per trip to meet the needs of themselves and their chick by efficiently searching an area for heterogeneously distributed prey. *aGannets* employ an arc in an attempt to locate prey, with the ability to switch to more sinuous and slow movements upon perceiving higher densities of prey, while limiting the distance they are flying from the colony to some extent.

### 4.5.2 Emergence

The movement patterns of *aGannet*s emerge from behavioural decisions made by gannets according to the prey density of their current location in combination with how successful they have been in the respective foraging trip thus far. We anticipate that upon inspection of foraging trips simulated for multiple individuals we may see some patterns typically represented at the population-level.

**IFSF simulation experiment modifications:** We imagine there will be emergence of individual foraging site fidelity depending on the hypotheses being used, and we anticipate that with increasing site fidelity we will see the emergence of a population distribution which overlaps with the prey area more effectively.

### 4.5.3 Learning

There is no learning included in this model which is implemented for subsequent foraging trips.

**IFSF simulation experiment modifications:** Memory was included in several different forms to allow gannets to refine foraging areas based on an approximation of foraging efficiency.

### 4.5.4 Sensing

When beginning a trip, it is assumed that *aGannet*s have a sense of which general direction to head in order to find profitable areas to forage, with knowledge of departure direction being implicitly assumed to have been gained prior to our simulated timescale. *aGannets* have the ability to sense fish through visual detection, and in areas with a higher density of prey gannets are more likely to begin ARS movement to try and forage for fish. They are aware of their how much prey they have captured during the current trip, and what their behaviour was during the previous time step. *aGannets* can also sense where land is within a certain distance so as to avoid flying over it.

### 4.5.5 Interaction

The only interaction between *aGannets* is indirect, through intraspecific competition, where prey is depleted for a certain cell when an *aGannet* has successfully foraged here, with prey being reset at the beginning of each new day in the model. However, if there is any effect of this on the system it is very weak, as prey are abundant, and simulations do not have over 100 individual *aGannets*. Interference competition (Lewis et al., 2001; Wakefield et al., 2013), wherein there is a reduction of prey availability close to the surface following multispecies feeding associations, as opposed to reduction in actual abundance of prey which is implied in the model, is likely to be an important factor in this system.

**IFSF simulation experiment modifications:** Public information was employed in several different ways, meaning that interaction could take the form of local enhancement or avoiding densely occupied areas.

### 4.5.6 Stochasticity

At each timestep in the model the step length for each individual is randomly drawn from a normal distribution for the relative movement mode, where the travel step length (Table S3) is used for outbound and inbound movement modes, and ARS step length (Table S3) for ARS movement mode. Turning angle for each step an *aGannet* is in ARS movement mode is drawn from a normal distribution (Table S3), whereas stochasticity is built into the submodels which calculate turning angles in outbound and inbound movement modes. Other stochastic processes include how long the individual spends at the colony between trips, which patch the *aGannet* picks as a target at the beginning of a trip, the size of the food consumed on a successful foraging attempt, and how long to rest after a fish has been caught.

### 4.5.7 Observation

Spatial coordinates of an *aGannet* at each time step during a foraging trip are recorded to a .csv file. These are then imported into R and transformed to be the same coordinate system (UTM) as the empirical data, thus allowing for direct comparison between simulated and observed data. This is key for spatial analysis such as inspection of fine scale movement, bearings, and overall length of foraging trips. Activity budgets are recorded directly from each simulation through tallies which count behaviours being exhibited as each simulation progresses and are recorded in a separate .csv file.

## 4.6 Initialisation

The model is initialised by loading raster files containing time spent foraging in grid cells representative of the North Sea which are then used to assign prey density by multiplying by assorted pre-determined constants to assign a level of prey (“high”/ “medium”/ “low”/ “critical”). Another raster is imported to demarcate cells which are land, which *aGannets* actively avoid. One hundred gannets are positioned at the colony and the first timestep in the model is the first minute of daylight at the beginning of the chick-rearing period under the assumption that all simulated gannets have a chick that hatches at the same time at the beginning of the run.

**IFSF simulation experiment modifications:** For each hypothesis 1000 gannets began from the colony during each simulation. Some hypotheses using memory required a burn-in period to simulate prior exploration of the environment so that individuals could begin the simulation with some experience of their environment.

## 4.7 Input data

There is no input data for the duration of the model run.

## 4.8 Submodels

Below we describe the submodels which are introduced in section 4.3 and are listed in the order that they are likely to be employed throughout the course of the model.

*decide-heading*

At the beginning of each trip an *aGannet* picks a random highly visited cell from empirical data which is at least 140km away from the colony. This is an important process in determining the bearing which an *aGannet* leaves the colony from, which is used in a subsequent submodel (“calculate-bearing”), but there is no requirement to reach this patch. This assumes that the agents in the model have a sense of the direction to head from the colony, which will have been gained during earlier life (Grecian et al., 2018; Votier et al., 2017) and fine-tuned for that particular year during the incubation period when they have been acting as central place foragers at the colony but with less constraints allowing them to make more exploratory trips (Lane et al., 2020). At the beginning of each trip a new target cell is picked by each simulated *aGannet*, and hence the often-repeatable departure bearing seen in empirical data (Patrick et al., 2014) associated with IFSF is not represented in this model.

**IFSF simulation experiment modifications:** For simulations with no memory (A, Table 1), at the beginning of each trip a random direction of travel was chosen due east of the colony (5^o^ - 140°) based on empirical observations of upper and lower limits of travel. Those including memory (B, C & D, Table 1) were based on the same mechanism but could recall previous targets to travel in the direction of, which we expand on in Section 5.1.

*enter-ARS?*

This submodel performs a stochastic process on the likelihood of switching from outbound to ARS movement mode, depending on the prey density of the cell that the *aGannet* currently occupies. This means that with increasing prey density, it is assumed that it is more likely that a gannet will spot this prey and decide to switch to ARS movement (Hamer et al., 2009).

Empirical evidence of such behaviours is difficult to obtain in the wild and thus understanding of predator-prey interactions of diving seabirds such as gannets is low. Captive experiments with cormorants (*Phalacrocorax auritus*) targeting juvenile rainbow trout (*Oncorhynchus mykiss*) showed varying prey densities had a strong influence on the success of the predator, where success linearly increased to an asymptote at higher densities (Enstipp et al., 2007). We extrapolate this to gannets visually detecting their prey and have characterised the relationship as an approximation of the type II curve (Figure S5) based on Holling’s functional response model (Holling, 1959).


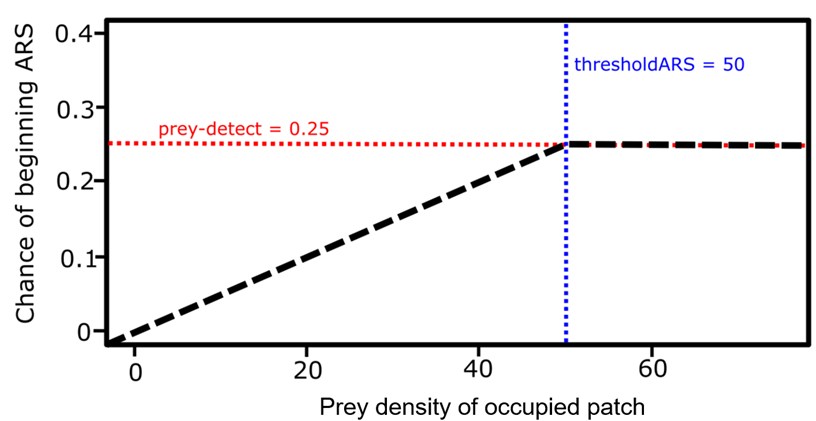
There are two unknown parameters in this submodel; *prey-detect* and *thresholdARS*. *Prey-detect* determines the chance of switching to ARS movement at any given timestep when an *aGannet* is in outbound travel, and if set to 0.25 will mean that there is never over 25% chance of beginning ARS (Figure S5). *thresholdARS* determines what prey density of the occupied patch needs to be exceeded (i.e. prey density of 50 in Figure S5 in order to have the maximum chance of detecting prey. The ability to detect prey decreases linearly in correspondence with decreasing prey density of a patch below *thresholdARS* (Figure S5).

Figure S5: Plot showing the relationship between chance of beginning ARS and prey density of the current patch which an aGannet may be on at any given moment with the global parameters of “thresholdARS” and “prey-detect” set to 50 and 0.25 respectively.

*Adjusting step length and turning angle*

These submodels draw randomly from a normal distribution of step length for outbound and inbound movement, and step length and turning angle for ARS movement. For approximations of step length distributions from empirical data see Figure S6 below. ARS step length was truncated at zero.


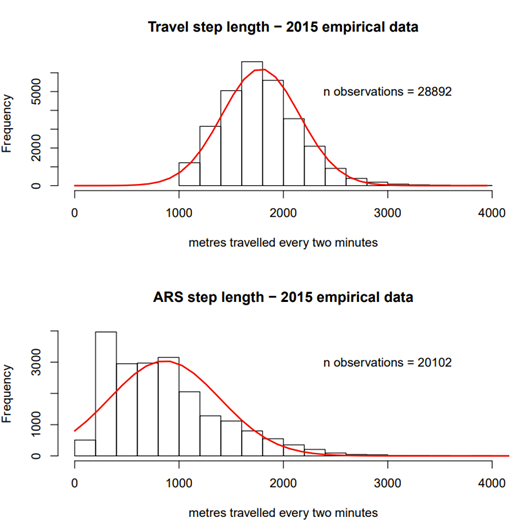


Figure S6: Step lengths of travel movement (top) and ARS movement (bottom) extracted from empirical data of adult gannets during chick rearing in 2015 at Bass Rock. Red curves show the corresponding normal distributions used for obtaining step lengths for respective movement modes.

*foraging-attempt*

At each timestep that an *aGannet* is in ARS movement this submodel is called, so there is the chance of catching a fish which is determined by the same functional response relationship and its associated parameters approximated for the “enter-ARS?” submodel (Figure S5). Thus, if an *aGannet* moves to another cell where prey density changes there can be an according response in the chance of catching prey at this timestep. If foraging has been successful an *aGannet’s* food intake will increase with the prey size being randomly drawn from a normal distribution derived from the literature (Table S3), and a Boolean variable will be switched to indicate to the following submodel that a rest is required.

*decide-rest-duration*

Inspection of empirical data indicates that the majority of rests are for a noticeably brief period of time, with the occasional longer rest and that these rests are largely nested within bouts of ARS movement (Figure S1). In Cape gannets (*Morus capensis*) it is suggested that rests which are commonly observed at the midpoint of the journey allow time to digest some food before returning home to feed the chick later in the day (Ropert-Coudert et al., 2004). Equivalent explanations have not been suggested in northern gannets, and as such we have decided to include a longer rest, drawn randomly from a normal distribution, towards the end of the trip when the threshold requirement for food intake has been met. Shorter rests after successful foraging attempts during ARS bouts earlier in the trip are randomly drawn from a gamma distribution (shape = 5, scale = 1).

*Calculate-bearing*

This submodel calculates the direction of outbound travel through an adjustment to the initial bearing which is recorded at the outset of the foraging trip based on the food intake of the gannet throughout the foraging trip. The equation used to calculate this adjustment is as follows:

$${Adjustment}_{i}= \frac{1}{(1 \div(1+ e^{- sqrt \left( (food-intake \right)-mean\left( food-intake \right)}) \times180+stoch}$$

Where *food-intake* is the total food that an *aGannet* has ingested (g) during that particular foraging trip. Stochasticity is introduced to the adjustment (Figure S7) through two coefficients applied so that there is a normal distribution of variation throughout the relationship (curve sd = 0.004, straight sd = 7). The calculated adjustment to the initial bearing, including stochastic variation, is then either added to or subtracted from the initial bearing according to whether the trip has been set as clockwise or anticlockwise, respectively.


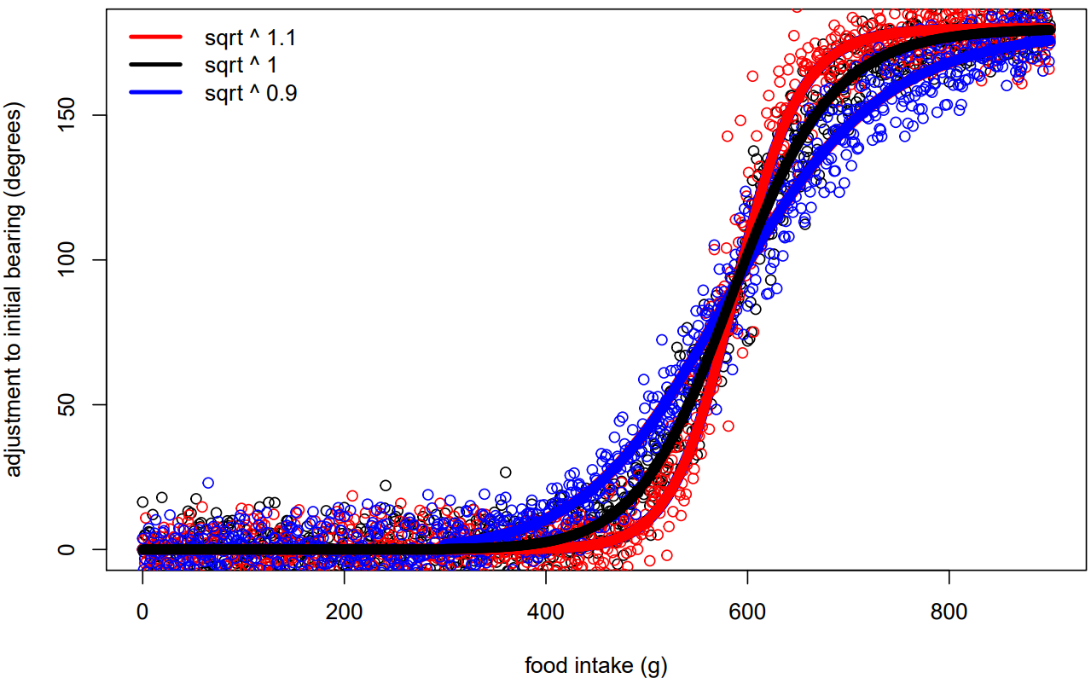


Figure S7: Logistic relationship of the adjustment to the original bearing made for food intake during a foraging trip where the different colour lines represent a different steepness implied in parameterisation tests with a visualisation of stochasticity around each line in the relevant colour.

The shallower the curve in this relationship (i.e. the blue curve in Figure S7), the more likely it is that the foraging trip will be more elliptical. Whereas when the curve is steeper (red curve in Figure S7), it is more likely that an *aGannet* will turn around sharply to face the colony, and the bearing travelling inwards will be very similar to the one which it travelled out on. We obtained the final relationship (Figure S7) to use through parameterisation procedures using the POM technique (see section 6 on parameterisation below).

*Calculate inbound*

For each step on inbound travel to the colony, this submodel is called. It uses a biased random walk, by including some bias towards facing the colony with a certain amount of stochasticity built into the turning angle. This represents what is seen empirically where it appears gannets know the general direction of the colony and travel towards it with some correction along the way (Figure S1). Then when birds are within 10 km of the colony it is assumed that they can see it or obtain directional cues from social information of colony-bound conspecifics and so travel straight back to the colony.

*Land avoidance*

This is a series of procedures adapted from code written previously and used to model Saimaa ringed seal (*Phoca hispida saimensis*) movements (Liukkonen et al., 2018). At each timestep during a foraging trip an *aGannet* will check if there is land in front of the direction it is facing (~5 km). If there is land ahead, the amount of land to the left and right of the individual is interpreted, and whichever direction there is more land in the gannet will decide to turn in the opposite direction so as to avoid this land.

*Decide length at colony*

This submodel decides how long an individual will spend at the colony after completing its foraging trip. It assumes that chick-rearing gannets have a partner that will be on a foraging trip if they themselves are at the colony. Thus, the waiting time at the colony will be approximately the same duration as the foraging trips. As the duration of the foraging trip will differ depending on the initial conditions of the model, this will need to be calculated throughout respective model runs. A list is stored and updated each time an *aGannet’s* trip is completed which retains the last 20 trips of any individual returning to the colony. The mean and standard deviation of this list of durations is then used to withdraw a number from a normal distribution on the *aGannet’s* return to the colony after its initial and subsequent foraging trips.

*Output files*

Depending on the output settings an *aGannet's* coordinates at each timestep are written so that movement analysis can be conducted in R, or a summary of times spent in different behaviours in each trip are written to a .csv for all individuals along with how many fish were caught on that trip.

# 5 Modelling conditions for IFSF simulation experiments

The following sections contain the biological background, core assumptions, and logical flow of the different modelled mechanisms used in our IFSF simulation experiments. These mechanisms are modifications of the foundational model. We aimed to represent each in a succinct and tractable way, to give the best chance at deciphering behavioural mechanisms that may underpin individual foraging consistency.

## 5.1 Private information

Mechanisms drawing on private information only influenced the departure direction of *aGannets* from the colony. Consequently, individuals had no obligation to reach a particular patch, and thus could engage in foraging opportunities whenever they arose on their outbound path (Wanless et al., 1990). As opposed to the foundational model, in which departure angle was based on selecting a bearing towards high density prey, departure angle choice for all simulations not deriving from previous memory was chosen at random between the bounds of 5-140°. This modification was made to capture the general direction of travel from the colony, inferred from empirical data, while avoiding the implication that any private information collected in prior trips was being drawn on.

### 5.1.1 Long-term memory

This mechanism was built on the assumption that gannets begin the chick-rearing period having conducted some exploration throughout the home range of the colony and have each settled on one departure bearing which if followed will probably lead to a profitable foraging location. It could be that this was learned earlier in life, in keeping with the “exploration-refinement” hypothesis where IFSF increases with age (Grecian et al., 2018; Votier et al., 2017), or during the immediately prior incubation period where adults experience less constraint and are known to forage further afield (Lane et al., 2020). The model did not differentiate between these two possibilities but simply included a long-term memory as described below.

To simulate this formation of memory, *aGannets* embarked on ten foraging trips, each in a random direction broadly eastwards from the colony (5^o^ to 140°, taken from empirical observations and avoiding travelling overland). Each *aGannet* recorded a daylight trip duration (i.e. total trip duration – any time spent resting on the water during overnight trips) in a list and its accompanying departure bearing. Once ten trips were completed the departure bearing with the lowest daylight trip duration was chosen and used for the subsequent simulation. Daylight trip duration was chosen over the time spent foraging per trip, as the constraints on parents to feed their chicks require them to return to the nest as quickly as possible (Hamer et al., 2007). Therefore, the selection on foraging areas is not driven purely by profitability of a patch, but also by its distance from the colony.

### 5.1.2 Short-term memory

Studies have shown that there is a minority of gannets that are inconsistent in departure direction and foraging area in consecutive trips (Hamer et al., 2001; Wakefield et al., 2015). These birds may have been switching to different foraging areas following a previously profitable area no longer proving so, known as “win-stay/lose-shift” (WSLS) foraging (Kamil, 1983). This switch could have stemmed from increased competition or lower prey availability, and most individuals in a population may adhere to similar mechanisms, implying that individuals are continually assessing the efficiency of foraging trips throughout the chick-rearing period. To implement this in the model, individuals kept a list of the daylight durations of the three most recent trips. At the beginning of a new foraging trip, if the three previous trips had all been increasing in duration, then the *aGannet* picked a new departure angle at random within the empirically determined confines (5 -140°). If the duration of this trip in a new direction proved to be longer than the previous two trips, then it drew another random departure angle (a so-called “sliding-window” mechanism); thus, if exploring a new foraging area was not proving profitable it moved on.

### 5.1.3 Combined memory

This mechanism assumes that individuals retain a long-term memory of multiple previously profitable departure directions, which can be switched between if a particular site is no longer proving profitable. As in the long-term memory mechanism described above *aGannets* each went through ten foraging trips prior to the simulation beginning. The three departure directions corresponding with the lowest daylight trip durations were stored in the individual’s memory. During the simulation, the continued use of a particular direction was subject to the mechanism used in short-term memory detailed above (5.1.2). As such, if the last three trips had been increasing in daylight trip duration, then the *aGannet* selected another departure direction stored in the long-term memory. Each *aGannet* begins the simulation on the departure direction corresponding with the lowest daylight trip duration and when switching would go in ascending order and cycle back to the earliest departure direction and continue as such.

## 5.2 Public information

For the following mechanisms we only considered the use of public information when at sea on a foraging trip (i.e. > 10 km from the colony) with justification provided in Section 2.2 above.

### 5.2.1 Local enhancement

Observed congregations of feeding gannets are consistent with local enhancement behaviour (Camphuysen, 2011), and bird borne cameras have recorded its prevalence in a closely related species (Tremblay et al., 2014). We modelled this mechanism so that *aGannets* in outbound movement mode could sense other *aGannets* exhibiting ARS movement, in a 270-degree field of vision up to 10 km away (Thiebault et al., 2014). We assumed that with increasing distance of conspecifics the likelihood of any *aGannet* pursuing this opportunity will decrease. We implemented this by having a 10% chance of pursuing a local enhancement opportunity if between 5-10 km away, and this increased to 20% if below 5 km at each timestep if an *aGannet* had any foraging conspecifics in its detectable range.

If the decision was made to pursue a local enhancement opportunity, the *aGannet* could sense the direction and distance of the conspecific and compute roughly how far it needed to travel to get to that patch. Therefore, it did not begin actively following that individual but headed towards the location where it was seen engaging in ARS behaviour. Once it reached the intended area it then resumed standard outbound behaviour and orientation. The functional response approximation was not altered, and hence the only way in which local enhancement could have increased the chance of an *aGannet* beginning ARS was if it had found a patch of higher prey density than those of the path it was previously on.

### 5.2.2 Competition

Regardless of whether increased trip duration at larger gannet colonies (Lewis et al., 2001; Wakefield et al., 2013) is driven by depletion of prey around the colony (Ashmole et al., 1971) and/or density-dependent disturbance of prey, it’s evident that some form of competition drives foraging gannets further afield when there are large numbers of conspecifics. Further to this, gannets have been observed to forage in relatively low densities (Camphuysen, 2011), which would indicate that they actively avoid high concentrations of conspecifics. We tested this in my model by programming *aGannets* to react to conspecifics in a contrasting fashion to local enhancement (5.2.1). When *aGannets* were in outbound travel they could sense the total number of conspecifics in a 270° field of vision up to 5km away. If there were more than two other *aGannets* in their vicinity, birds would not begin ARS movement even if situated on a relatively dense patch of prey. This effectively drove *aGannets* to travel further from the colony to try and find a less busy patch.

### 5.2.3 Combination of local enhancement and competition

This mechanism was developed under the assumption that local enhancement and competition may act in concurrence. In this hypothesis, *aGannets* were only able to use local enhancement through sensing foraging conspecifics if they were not experiencing competition as defined above (5.2.2). Thus, individuals had to find a relatively sparsely populated area before the possibility of beginning ARS movement or pursuing local enhancement opportunities.

# 6 Parameterisation

## 6.2 Movement model

### 6.2.1 Methods

The majority of the parameter values in this study were given values derived either from literature or empirical data (Table S3). However, a few important parameters are unknown, so we devised a series of parameter estimation procedures, using POM to obtain unknown parameters which is sometimes referred to as “inverse determination” (Grimm & Railsback, 2012). This was a three-step procedure in which one step was completed before beginning the next:

1. Fine scale movements: Before looking at broader scale patterns we ensured that fine scale movements were being modelled well by visual comparison of distribution of step lengths and turning angles of simulated outbound, ARS and inbound movements against those from empirical data (Table S1). Here one hundred simulated trips were run with different stochasticity coefficients until they matched what was seen in one hundred randomly selected empirical foraging trips. Simulated outbound and inbound data were compared separately against pooled travel fine scale movement data (i.e. outbound and inbound are not delineated), as they are the result of different submodels thus requiring separate inspection.
2.
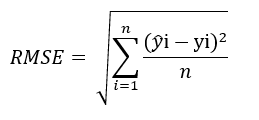
Functional response parameters: POM was used to identify what combination of three interacting parameters (*prey-density*, *prey-detection*, *thresholdARS*) resulted in emergence of trip durations (h) most similar to what was observed in the empirical data (Table S1). The different parameter values used were: (i) four levels of the landscape attribute *prey-density* (critical/low/medium/high) achieved through heuristic determination by using a coefficient to multiply the time spent foraging in particular cells in order to cover the full scope of foraging ranges when all other parameters were kept at a constant intermediate level, (ii) *prey-detection* proved to be very sensitive on pilot experiments, thus it was given high resolution with values in the range 0.05 – 0.3, with 0.025 intervals. (iii) *ThresholdARS* values were in the range of 20 – 100, with intervals of 20. For each of the resultant 220 parameter combinations we ran the model and extracted 100 simulated foraging trips at random. 100 trip durations were extracted from the empirical data for comparison which were used as the observed values for calculation of the root mean square error (RMSE) scores in order to evaluate the agreement between the observed and predicted values:

Through grouping the lowest score and visual inspection of plots we ascertained the best fitting parameter combination and use this going forward.

1. Logistic relationship between food intake and adjustment to initial bearing: The final procedure for parameterisation was designed to capture broad scale movement patterns, i.e. how elliptical or linear the trip was. We varied the steepness coefficient of the curve in the relationship in 3 levels (steep/normal/shallow). With increasing steepness of the curve (Figure S7) comes lower possibility to withdraw from the curve on the transition from 0 - 180° as food intake is increasing in any particular foraging trip, and hence the likelihood of having a more directed (less elliptical) foraging trip trajectory. Each of these three parameter levels was run in a simulation and 100 trips were exported for spatial analysis in R where we calculated the linearity index (Section 3.2) for each trip, where a value of 1 would indicate a perfectly directed trip to a maximal point and back, with increasing values indicating deviation from this. We then plotted the linearity probability densities for respective simulations against empirical data from 100 trips from two different years (2012, 2016), and it was apparent that different years showed considerable variation in shapes, from which we then visually assessed which parameter level would be best. Another pattern described in section 3.2, bearing difference between initial departure angle and maximum point, was considered but did not provide any further insights beyond those obtained from assessment with the linearity index and visual assessment of the shapes trips, so was not included in this process.

### 6.2.2 Results

#### 6.2.2.1 Fine scale movements

After iteratively running different stochasticity levels, step lengths and turning angles and assessing outputs the final parameters were decided for fine scale movement which produced the step lengths and turning angles seen in Figures S8 and S9, respectively. The modelled step lengths for travel (outbound and inbound movement modes) were 0.91 ± 0.17. Those for ARS were = 0.429 ± 0.23. The final values for stochasticity coefficients which influenced turning angles were as follows: curve stoch sd = 0.004, straight stoch sd = 8, calculate inbound stoch sd = 10, setting for sd of ARS turning angle = 46.


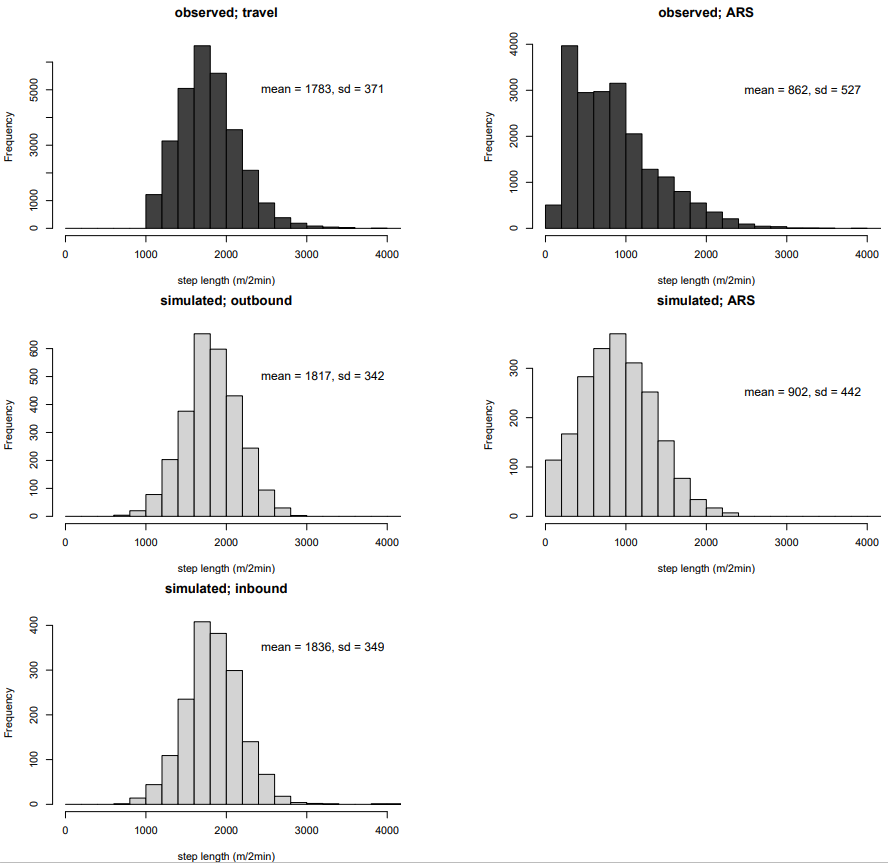


Figure S8: Distribution of step lengths for travel are on the left, ARS are on the right. The top row of darker grey plots are the observed distributions from empirical data, with the lighter grey plots below from simulated data.


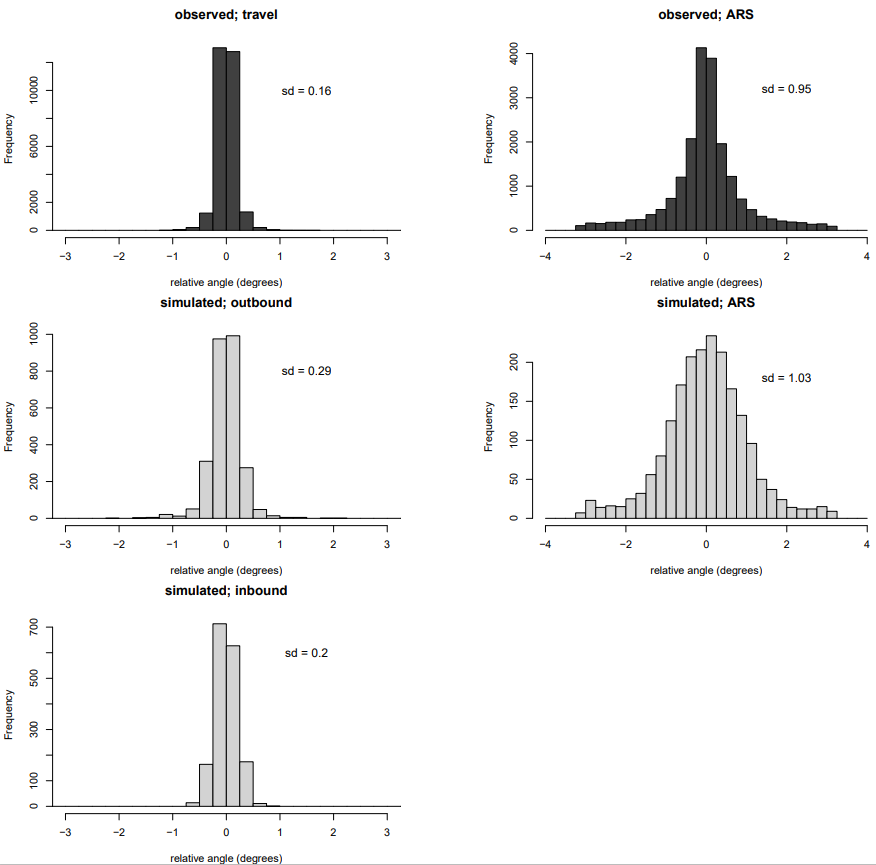


Figure S9: Distribution of turning angles for travel are on the left, ARS are on the right. The top row of darker grey plots are the observed distributions from empirical data, with the lighter grey plots below from simulated data.

*6.2.2.2 Functional response*

There was high variability in the outputs resulting from the 220 different parameter combinations with considerable overlap between their ability to reproduce the chosen empirical pattern (Figure S10). This was predictable given the interactive nature in dictating the modelled functional response of the three parameters tested. On further inspection it was clear that beyond the lowest 20 scores (i.e. the top scoring combinations) the pattern being inspected was not well captured. Therefore, we selected from the top 20 scores (Table S5) by plotting the trip durations against the empirical data (Figure S11) and visually deduced the three best fitting parameter combinations according to the mean and variance of the data (represented by yellow and orange highlighted parameter combinations in Table S5 and Figure S11). We then ran each of these combinations in turn in the model through visualising in the interface and there was not much perceivable difference so settled on the 10^th^ ranked parameter combination, which is indicated by orange in Table S5 and Figure S11, due to median and interquartile range being well represented (Figure S11).


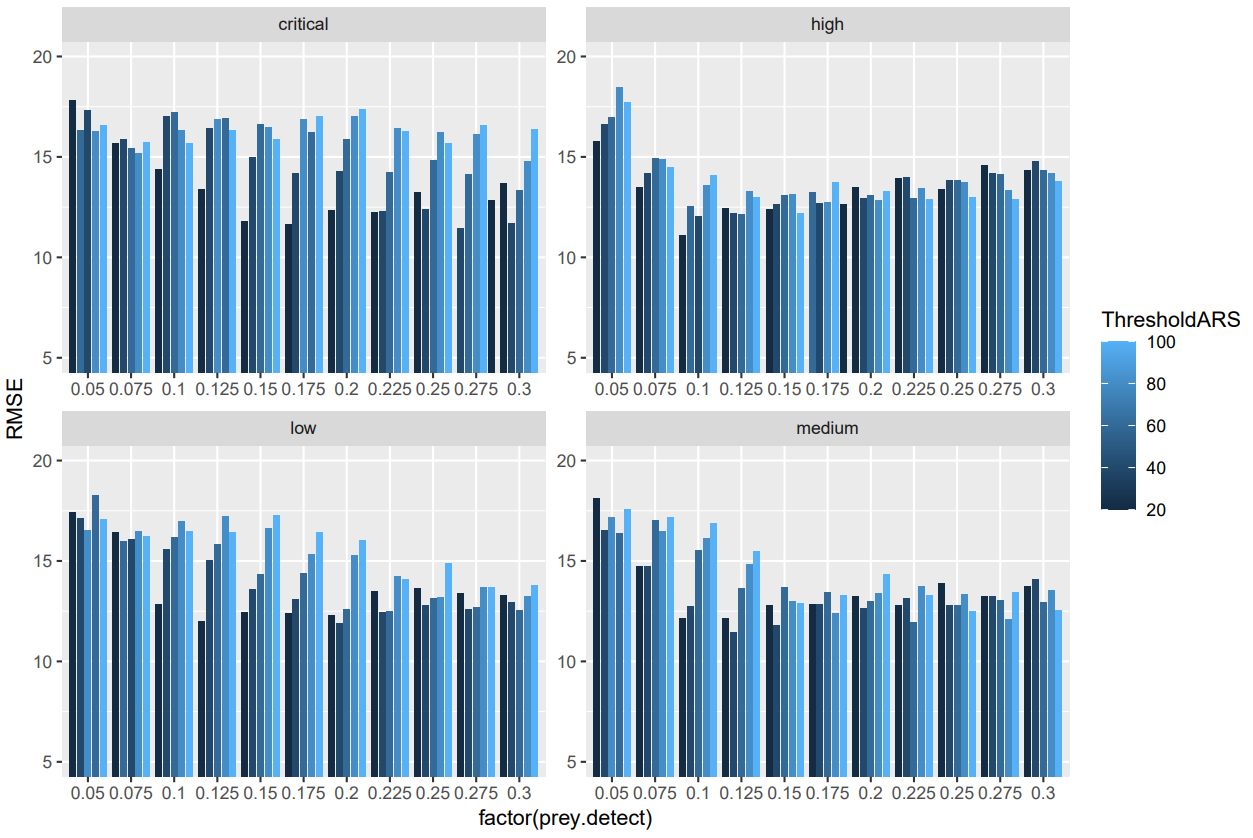


Figure S10: Bar plots showing root mean square error (RMSE) scores of parameterisation tests for three interacting parameters which dictate foraging behaviour through the functional response approximation process: (i) “Prey density” is related to the overall availability of prey in the landscape and is assigned four levels (high, medium, low, critical) which are shown in the four windows (ii) “ThesholdARS” dictates how the approximated type II functional response asymptotes and was assigned five different values and is displayed by the colour of the bar, and (iii) “prey detect” which dictates the chance of beginning ARS was tested at 11 increments, which are displayed on the x-axes.

Table S5: Rankings of top 20 (i.e. lowest) root mean square error (RMSE) scores from our functional response parameterisation. The coloured rows indicate the top 3 combinations picked from visual assessment of trip durations (Figure S11).

| RMSE | Rank | prey.density | prey.detect | ThresholdARS |
| --- | --- | --- | --- | --- |
| 11.088 | 1 | high | 0.1 | 20 |
| 11.42293 | 2 | critical | 0.275 | 40 |
| 11.43108 | 3 | medium | 0.125 | 40 |
| 11.66516 | 4 | critical | 0.175 | 20 |
| 11.70821 | 5 | critical | 0.3 | 40 |
| 11.78626 | 6 | medium | 0.15 | 40 |
| 11.81091 | 7 | critical | 0.15 | 20 |
| 11.87579 | 8 | low | 0.2 | 40 |
| 11.93222 | 9 | medium | 0.225 | 60 |
| 11.98523 | 10 | low | 0.125 | 20 |
| 12.01943 | 11 | high | 0.1 | 40 |
| 12.10548 | 12 | medium | 0.275 | 80 |
| 12.1196 | 13 | high | 0.125 | 60 |
| 12.1619 | 14 | medium | 0.125 | 20 |
| 12.16357 | 15 | medium | 0.1 | 20 |
| 12.16982 | 16 | high | 0.15 | 100 |
| 12.19935 | 17 | high | 0.125 | 40 |
| 12.22273 | 18 | critical | 0.225 | 20 |
| 12.27497 | 19 | low | 0.2 | 20 |
| 12.2784 | 20 | critical | 0.225 | 40 |


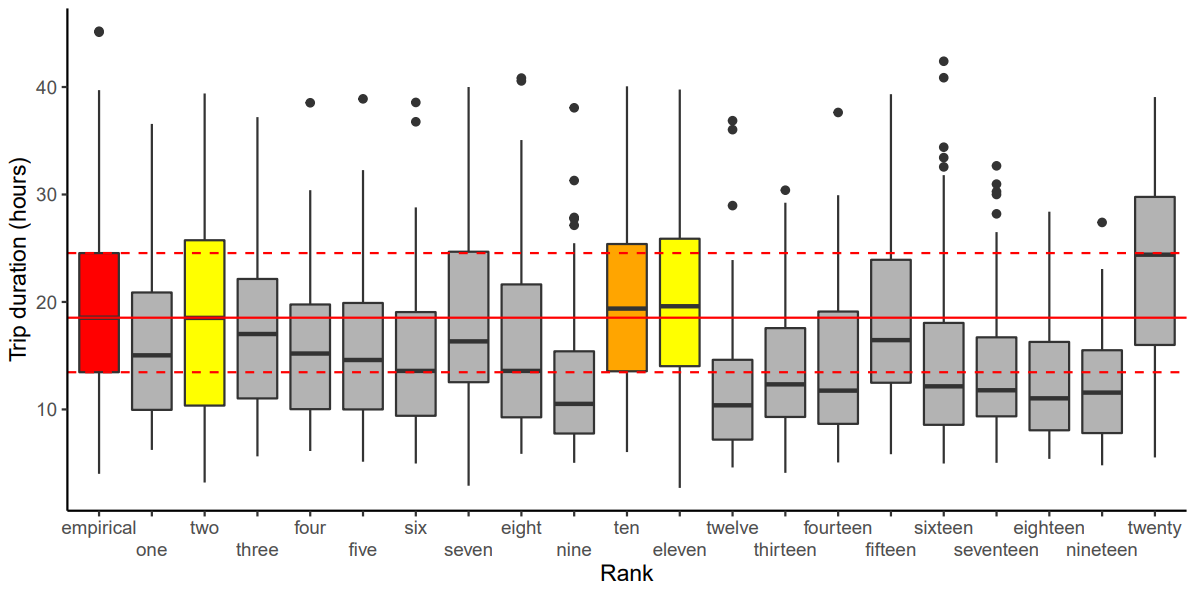


Figure S11: Trip durations (hours) of the lowest 20 RMSE scores out of 220 parameter combinations. The red box on the far left is from empirical observations with solid red line indicating the empirical median, and upper and lower red dashed lines indicate the respective quartiles. The other coloured plots indicate the top 3 selected parameter combinations which were run on the model interface, with orange being the final one selected for the model going forwards.

#### 6.2.2.3 Logistic relationship between food-intake and adjustment to initial bearing

The different shapes of logistic curve tested (steep/normal/shallow) showed some variation in their emergent linearity index, where the shallow curve resulted in less linear trips, as intended. “Steep” and “normal” parameter levels produced trips with almost identical linearity, whereas the “shallow” parameter level showed noticeably less linearity (Figure S12). However, the variation between parameter levels was small in comparison to that seen in the interannual variation in empirical data (Figure S12). All tested parameter levels were on average more linear than empirical trips from 2012, and less linear than trips from 2016, and therefore all tests fell within the range of possibility. After visualising trip trajectories from different parameter levels, it was decided not to use the “shallow” parameter level as it had the tendency to produce unrealistic foraging trip trajectories, and so we opted for the intermediate “normal” logistic curve going forwards.

Figure S12: Plots of the linearity index of trips from outputs of three tested parameter levels (steep/normal/shallow) for the logistic curve dictating the relationship between food intake and adjustment to initial bearing, compared with the linearity index of empirical trips from 2012 (left) and 2016 (right).


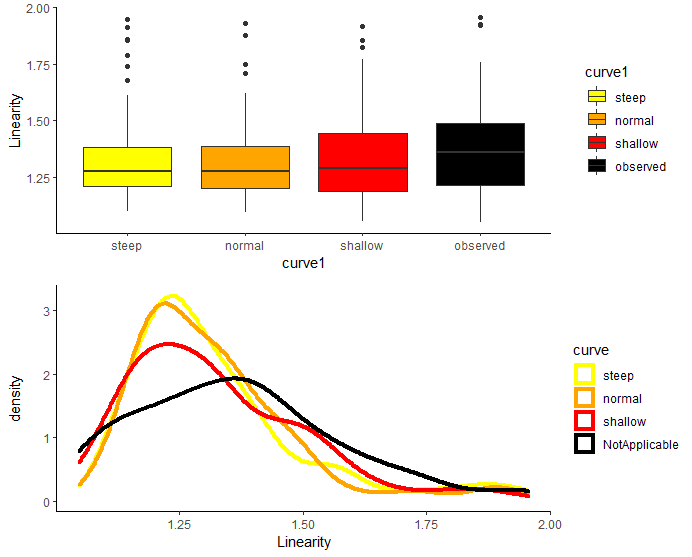

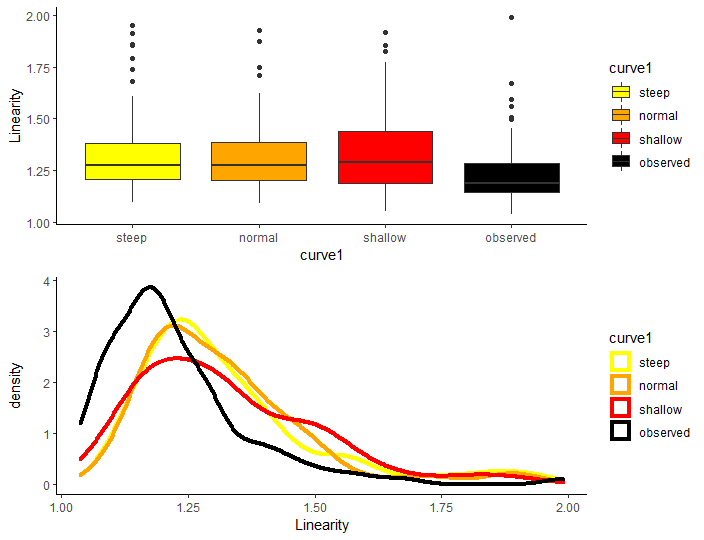


2012 data *n* trips = 100 2016 data *n* trips = 100

## 6.3 IFSF simulation experiments

### 6.3.1 Methods

We attempted to quantify parameters from the literature where we could for the different resource localisation mechanisms (see Table S6). However, given that such behavioural parameters are hard to determine through empirical studies we first sought expert opinion and discussion on the potential nature of the modelled mechanisms. For mechanisms using public information (local enhancement and competition) we ran simulations while observing the NetLogo interface (Figure S13) and observed outputs while varying parameters (e.g. number of birds to avoid during competition) with the goal of it having a perceivable influence on foraging dynamics without being overbearing.

Table S6: Parameters implemented for the relevant hypotheses in different simulation experiments.

| **Information source** | **Parameter** | **Relevant hypotheses** | **Value** | **Source** |
| --- | --- | --- | --- | --- |
| **Public** | Reaction distance | Local enhancement | Up to 10 km | (Thiebault et al., 2014) |
|  | Likelihood of venturing towards foraging conspecific | Local enhancement | 20% if < 5 km away; 10% if >= 5 km away | Derived heuristically |
|  | Field of vision | Local enhancement and competition | 270 degrees | Derived heuristically |
|  | Competition conspecific threshold | Competition | 2 conspecifics (equivalent to >100 gannets when scaled up to actual population size) | Derived heuristically |
|  | Distance conspecifics are considered competitors | Competition | Up to 5 km | Derived heuristically |
| **Private** | Number of trips used to ascertain my departure bearing (long-term)/bearings (combined) | Long-term and combined memory | 10 foraging trips | Derived heuristically |
|  | Number of departure bearings stored in memory following selection based on duration | Long-term and combined | Long-term = 1;  Combined = 3 | Derived heuristically |
|  | Number of immediately prior foraging trips assessed to decide whether to change direction | Short-term and combined memory | 3 foraging trips | Derived heuristically |

###
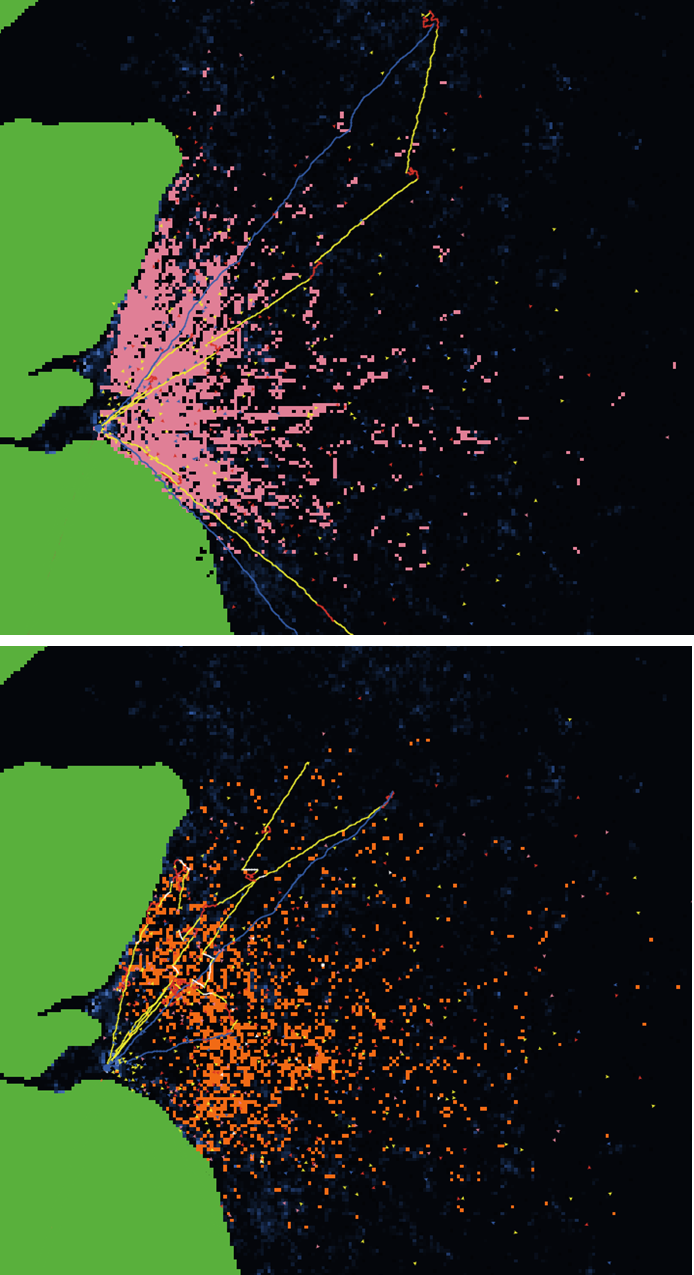
6.3.2 Results

Figure S13: Screenshots of the interface of our NetLogo model when calibrating for competition levels (top) and local enhancement (bottom). The green area is land, and cells with various colours of blue/black indicate prey density (lighter blue = higher density). Small, coloured arrows represent other aGannets at sea on foraging trips with colours representing their current movement mode (yellow = outbound, red = ARS, blue = inbound, white = local enhancement, bottom only), with a subset of individuals displaying their tracks. Pink cells (top) indicate areas in which aGannets have experienced competition, the majority of which are close to the colony as is perceivably the case in reality. Orange cells (bottom) indicate areas which aGannets have moved to when pursuing local enhancement opportunities.

# 7 Sensitivity analysis

## 7.1 Methods

To assess the sensitivity of the model’s output to changes in parameter values we performed a local sensitivity analysis. This was achieved by running simulations with singular, successive perturbation of a subset of model parameters. This subset was composed of the majority of important parameters (Table S4), including those contributing to stochasticity, with the exclusion of those relating to step lengths and turning angles for different movement modes as this would have resulted in the model’s deviation from representative movement patterns. Each parameter was varied by a standard range of ±10% in turn, while all other parameters were maintained at the level discerned through the parameterisation process, and trip durations (h) of 1000 trips from 100 individuals were withdrawn from the simulation. The difference in mean trip duration (h) was then expressed as a percentage difference from that of the baseline trip duration from a model run with no parameter perturbation.

## 7.2 Results

Eight parameters were varied in the local sensitivity analysis. Out of the 16 simulations (each parameter varied ± 10%) differences in outputs compared to the baseline model were only exceeded on three occasions (Figure S14). Changes in model outputs were the most sensitive to the parameter which dictates the chance of detecting prey in the model (*prey detect*) The other two parameters which contribute to the functional response (*ThresholdARS* and *prey level*) did not have as much influence on model outputs. The duration of short rests and fish size (g) had the lowest influence of all tested parameter in influencing model output (Figure S14).


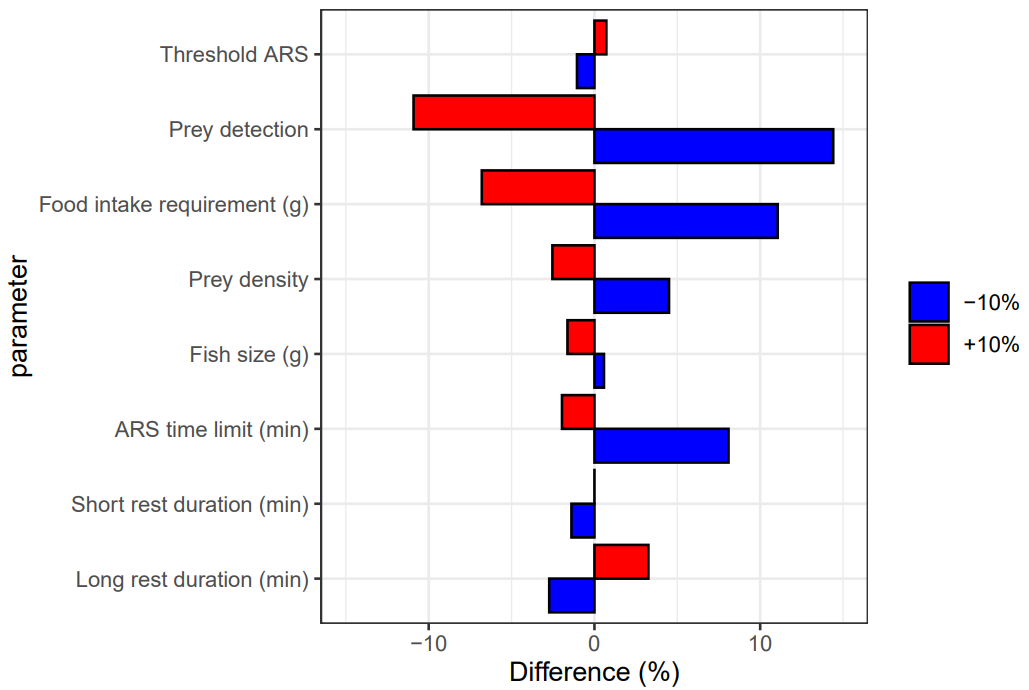


Figure S14: Sensitivity analysis of trip duration (h) to single and sequential variation (±10%) of parameters listed on the y-axis where differences are expressed as percentage difference to a baseline where all parameters were kept at their default level.

# 8 Evaluation

## 8.1 Movement model

### 8.1.2 Methods

A final evaluation of the model was achieved through side-by-side visual comparison of 3 random modelled trips with 3 randomly picked empirical trips and inspection to see if the correct activity budgets were being observed. We also observed potential population patterns of this model, although not the focus of this model, by side-by-side plots of 100 trips from modelled and empirical data on the same plot to see if any population-level patterns were emerging.

### 8.1.3 Results

When comparing a subset of simulated foraging trips from the fully parameterised model with empirical foraging tracks (Figure 2) it is clear that the modelled trips have captured many key attributes of the natural system, in terms of both size and shape. There was considerable variation in the trip metrics which reflected the variation seen in empirical data. The resulting arc from “trap-line” trajectory results in some trips being more elliptical, while others were more direct. The relationship between food intake and adjustment to initial bearing has allowed for trips where foraging can take place once heading back in the general direction of the colony. However, it is still possible to differentiate simulated tracks from empirical due to the relatively straight travel between ARS zones (Figure 2), which is an artefact of the bearing adjustment being a product of food intake which can only occur during ARS movement.

To detect any emergent patterns at the population-level we plotted 100 simulated trips to compare alongside 100 empirical trips (Figure S15). Despite not being the model’s intended purpose, it is clear that population patterns are being reproduced to some extent, with the accessible space being exploited in a similar fashion. The model shows that most foraging is taking place to the northeast, a consistent pattern in gannets foraging from Bass Rock, and this can be explained by the modelled individuals being informed about which directions have profitable foraging.


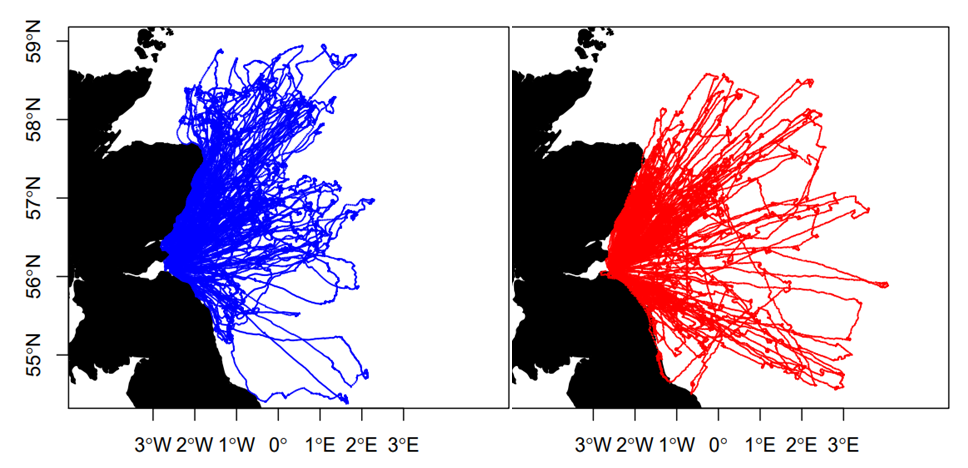


Figure S15: Comparison of 100 simulated foraging trips (right) with 100 empirical foraging trips (left).

When the activity budgets of foraging trips from the final model’s outputs are compared against empirical data (Figure S16) We found the proportion of time spent in ARS movement was representative, but there was a lower proportion of time spent resting, and a higher proportion of time spent in travelling movement modes (“outbound” and “inbound”). It is unlikely that this had much influence on foraging trip trajectory, but this is something that should be addressed in future developments of this model.


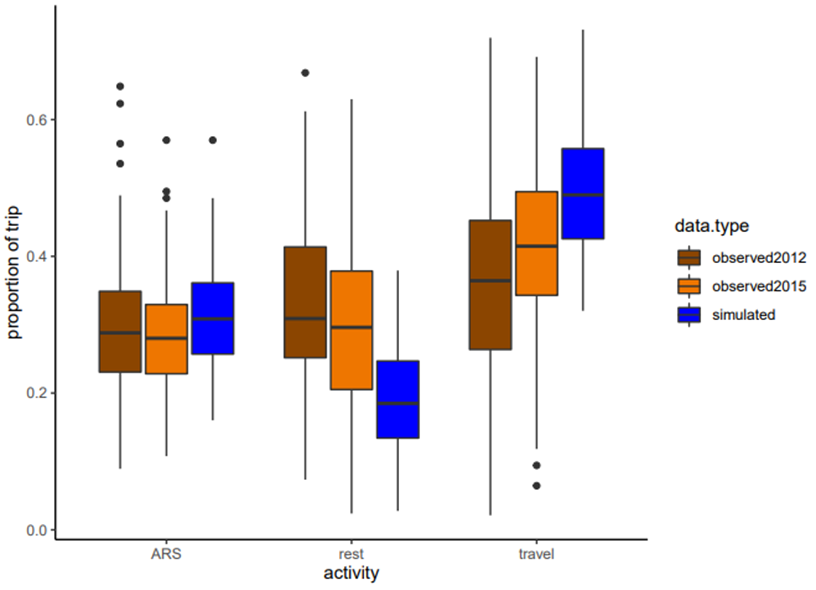


Figure S16: Proportion of trip spent in different activities (travel/ARS/rest) for model outputs compared with empirical data.

## 8.2 IFSF simulation experiments results

Table S7: Results of the different patterns used for evaluation of model outputs against empirical data for three consecutive trips per individual. For the conditions of the hypotheses refer to Table 1 (main text). The simulation value(s) closest to the empirical value for each respective pattern is highlighted with bold type and underlined. Significance level of relevant test for repeatability of trip duration and BA against a null distribution: ° p < 0.1; * p < 0.05; ** p < 0.01; *** p < 0.001.

| **Hypothesis** | **Evaluation pattern** | | | | | | | | | |
| --- | --- | --- | --- | --- | --- | --- | --- | --- | --- | --- |
|  | IFSF | | | | | Foraging efficiency | | | | |
|  | Repeatability of daylight trip duration | Repeatability of departure angle | Mean BA of successive trips by each individual | Mean absolute error (MAE) of BA compared with observed (rank) | Kolmogorov-Smirnov (KS) test of BA distributions – *D* statistic (p-value) | Schoener’s D index of overlap and rank | Index of % overlap with empirical UD | | Daylight trip duration mean & sd (hours) | Maximum distance mean & sd (km) |
|  |  |  |  |  |  |  | 95% UD | 50% UD |  |  |
| *Observed*  (n=98) | 0 ± 0.03 | 0.54 ± 0.06 | 0.47 (0.12, 0.99) | *-* | - | 0.648 | 1.298 | 0.219 | 16.4 ± 8.3 | 198 ± 103 |
| A1  (n=100) | 0.01 ± 0.03 | 0.04 ± 0.02 | 0.27 (0.02, 0.86) | 0.20 (13) | 0.421 (<0.001) | 0.189 | 1.033 | 0.080 | 13.5 ± 5.3 | 178 ± 99 |
| A2 | 0.04 ± 0.04 | 0.02 ± 0.02 | 0.26 (0.03, 0.82) | 0.21 (14) | 0.464 (<0.001) | 0.187 | 0.969 | 0.081 | 14.0 ± 5.7 | 176 ± 102 |
| A3 | 0 ± 0.04 | 0.05 ± 0.02 | 0.22 (0.02, 0.62) | 0.24 (15) | 0.586 (<0.001) | 0.191 | 0.963 | 0.093 | 14.7 ± 5.7 | 206 ± 103 |
| A4 | 0.05 ± 0.05 | 0.08 ± 0.02 | 0.22 (0.00, 0.74) | 0.25 (16) | 0.556 (<0.001) | 0.193 | 0.944 | 0.100 | 14.5 ± 5.7 | 198 ± 104 |
| B1 | 0.12 ± 0.07 | 0.99 ± 0 | 0.58 (0.21, 0.88) *** | 0.13 (12) | 0.301 (<0.001) | 0.191 | 1.083 | 0.099 | 12.4 ± 5 | 161 ± 91 |
| B2 | 0.15 ± 0.06 | 0.98 ± 0 | 0.57 (0.26, 0.92) *** | 0.11 (11) | 0.270 (0.001) | 0.199 | 1.163 | 0.086 | 12.4 ± 5.1 | 151 ± 91 |
| B3 | 0 ± 0.03 | 0.99 ± 0 | 0.46 (0.13, 0.84) *** | 0.05 (6) | 0.155 (0.161) | 0.194 | 0.996 | 0.114 | 14.4 ± 5.6 | 202 ± 104 |
| B4 | 0.07 ± 0.06 | 0.98 ± 0  (0.44) | 0.48 (0.15, 0.92) *** | 0.03 (1) | 0.110 (0.529) | 0.195 | 1.081 | 0.121 | 14.2 ± 5.4 | 191 ± 99 |
| C1 | 0.17 ± 0.06 | 0.72 ± 0.01 | 0.50 (0.13, 0.92) *** | 0.05 (5) | 0.142 (0.241) | 0.197 | 1.039 | 0.103 | 13.1 ± 5.7 | 172 ± 100 |
| C2 | 0.2 ± 0.07 | 0.72 ± 0.01 | 0.50 (0.06, 0.93) *** | 0.05 (4) | 0.180 (0.068) | 0.199 | 1.076 | 0.102 | 13.2 ± 5.7 | 166 ± 98 |
| C3 | 0 ± 0.03 | 0.79 ± 0.01 | 0.42 (0.02, 0.83) *** | 0.04 (2) | 0.156 (0.157) | 0.192 | 1.043 | 0.089 | 14.8 ± 5.6 | 207 ± 102 |
| C4 | 0.04 ± 0.05 | 0.77 ± 0.01 | 0.42 (0.11, 0.80) *** | 0.04 (3) | 0.165 (0.117) | 0.197 | 1.094 | 0.120 | 14.2 ± 5.7 | 193 ± 105 |
| D1 | 0.08 ± 0.05 | 0.84 ± 0.01 | 0.52 (0.14, 0.96) *** | 0.06 (9) | 0.180 (0.068) | 0.198 | 1.069 | 0.101 | 12.9 ± 5.2 | 166 ± 88 |
| D2 | 0.03 ± 0.05 | 0.83 ± 0.01 | 0.55 (0.06, 0.92) *** | 0.10 (10) | 0.227 (0.010) | 0.194 | 1.034 | 0.092 | 12.5 ± 5.2 | 155 ± 93 |
| D3 | 0.06 ± 0.05 | 0.69 ± 0.01 | 0.40 (0.00, 0.88) *** | 0.05 (7) | 0.155 (0.161) | 0.204 | 1.133 | 0.122 | 14.7 ± 5.8 | 208 ± 108 |
| D4 | 0 ± 0.04 | 0.75 ± 0.01 | 0.40 (0.08, 0.81) *** | 0.06 (8) | 0.198 (0.034) | 0.198 | 1.104 | 0.121 | 14.6 ± 5.9 | 202 ± 108 |

Table S8: Results of the different patterns used for evaluation of model outputs against empirical data for six consecutive trips per individual. For the conditions of the hypotheses refer to Table 1 (main text). The simulation value(s) closest to the empirical value for each respective pattern is highlighted with bold type and underlined. Significance level of relevant test for repeatability of trip duration and BA against a null distribution: ° p < 0.1; * p < 0.05; ** p < 0.01; *** p < 0.001.

| **Hypothesis** | **Evaluation pattern** | | | | | | | | | |
| --- | --- | --- | --- | --- | --- | --- | --- | --- | --- | --- |
|  | IFSF | | | | | Foraging efficiency | | | | |
|  | Repeatability of daylight trip duration | Repeatability of departure angle | Mean BA of successive trips by each individual | Mean absolute error (MAE) compared with observed & rank | Kolmogorov-Smirnov (KS) test of BA distributions – *D* statistic (p-value) | Schoener’s D index of overlap and rank | Index of % overlap with empirical UD | | Daylight trip duration mean & sd (hours) | Maximum distance mean & sd (km) |
|  |  |  |  |  |  |  | 95% UD | 50% UD |  |  |
| *Observed*  (n=33) | 0.11 ± 0.06 | 0.68 ± 0.07 | 0.53 (0.26, 0.99) | - | - | 0.516 | 1.213 | 0.195 | 13.8 ± 7.1 | 162 ± 97 |
| A1  (n=100) | 0 ± 0.02 | -0.01 ± 0.01 | 0.25 (0.09, 0.45) | 0.27 (14) | 0.749 (<0.001) | 0.203 | 1.064 | 0.075 | 13.4 ± 5.3 | 177 ± 97 |
| A2 | 0 ± 0.02 | -0.01 ± 0.01 | 0.28 (0.09, 0.66) | 0.23 (13) | 0.638 (<0.001) | 0.201 | 0.995 | 0.072 | 13.6 ± 5.5 | 173 ± 99 |
| A3 | 0.01 ± 0.02 | 0.04 ± 0.01 | 0.22 (0.08, 0.46) | 0.30 (15) | 0.849 (<0.001) | 0.207 | 0.992 | 0.091 | 14.7 ± 5.8 | 201 ± 104 |
| A4 | 0.01 ± 0.02 | 0.04 ± 0.01 | 0.21 (0.06, 0.44) | 0.30 (16) | 0.839 (<0.001) | 0.208 | 0.986 | 0.093 | 14.2 ± 5.7 | 194 ± 105 |
| B1 | 0.07 ± 0.03 | 0.99 ± 0 | 0.55 (0.27, 0.82) *** | 0.05 (1) | 0.233 (0.115) | 0.211 | 1.149 | 0.106 | 12.7 ± 5.1 | 164 ± 90 |
| B2 | 0.17 ± 0.04 | 0.98 ± 0 | 0.57 (0.30, 0.86) *** | 0.08 (8) | 0.326 (0.008) | 0.215 | 1.149 | 0.096 | 12.8 ± 5.5 | 159 ± 96 |
| B3 | 0.05 ± 0.03 | 0.99 ± 0 | 0.46 (0.25, 0.74) *** | 0.07 (6) | 0.245 (0.085) | 0.208 | 1.047 | 0.112 | 14.3 ± 5.9 | 199 ± 107 |
| B4 | 0.08 ± 0.03 | 0.99 ± 0 | 0.49 (0.19, 0.79) *** | 0.05 (2) | 0.193 (0.278) | 0.217 | 1.123 | 0.115 | 14.1 ± 5.7 | 191 ± 101 |
| C1 | 0.11 ± 0.04 | 0.72 ± 0.01 | 0.44 (0.17, 0.89) *** | 0.08 (7) | 0.237 (0.102) | 0.209 | 1.021 | 0.107 | 13.5 ± 5.7 | 179 ± 103 |
| C2 | 0.11 ± 0.04 | 0.64 ± 0.01 | 0.46 (0.19, 0.80) *** | 0.06 (4) | 0.185 (0.316) | 0.208 | 1.068 | 0.090 | 13.3 ± 5.4 | 167 ± 96 |
| C3 | 0.02 ± 0.02 | 0.69 ± 0.01 | 0.39 (0.14, 0.71) *** | 0.12 (10) | 0.335 (0.006) | 0.209 | 1.030 | 0.095 | 14.8 ± 5.9 | 208 ± 107 |
| C4 | 0.07 ± 0.03 | 0.69 ± 0.01 | 0.40 (0.15, 0.77) *** | 0.11 (9) | 0.308 (0.013) | 0.213 | 1.153 | 0.113 | 14.2 ± 5.6 | 192 ± 102 |
| D1 | 0.1 ± 0.03 | 0.71 ± 0.01 | 0.45 (0.15, 0.76) *** | 0.06 (5) | 0.237 (0.102) | 0.211 | 1.115 | 0.102 | 13.2 ± 5.6 | 172 ± 100 |
| D2 | 0.04 ± 0.03 | 0.69 ± 0.01 | 0.46 (0.19, 0.88) *** | 0.05 (3) | 0.177 (0.367) | 0.212 | 1.158 | 0.090 | 12.7 ± 5.3 | 158 ± 93 |
| D3 | 0.07 ± 0.03 | 0.63 ± 0.01 | 0.39 (0.14, 0.76) *** | 0.12 (11) | 0.337 (0.005) | 0.222 | 1.185 | 0.118 | 14.1 ± 5.6 | 197 ± 103 |
| D4 | 0.02 ± 0.02 | 0.64 ± 0.01 | 0.38 (0.13, 0.68) *** | 0.14 (12) | 0.407 (<0.001) | 0.225 | 1.179 | 0.121 | 13.9 ± 5.5 | 187 ± 99 |


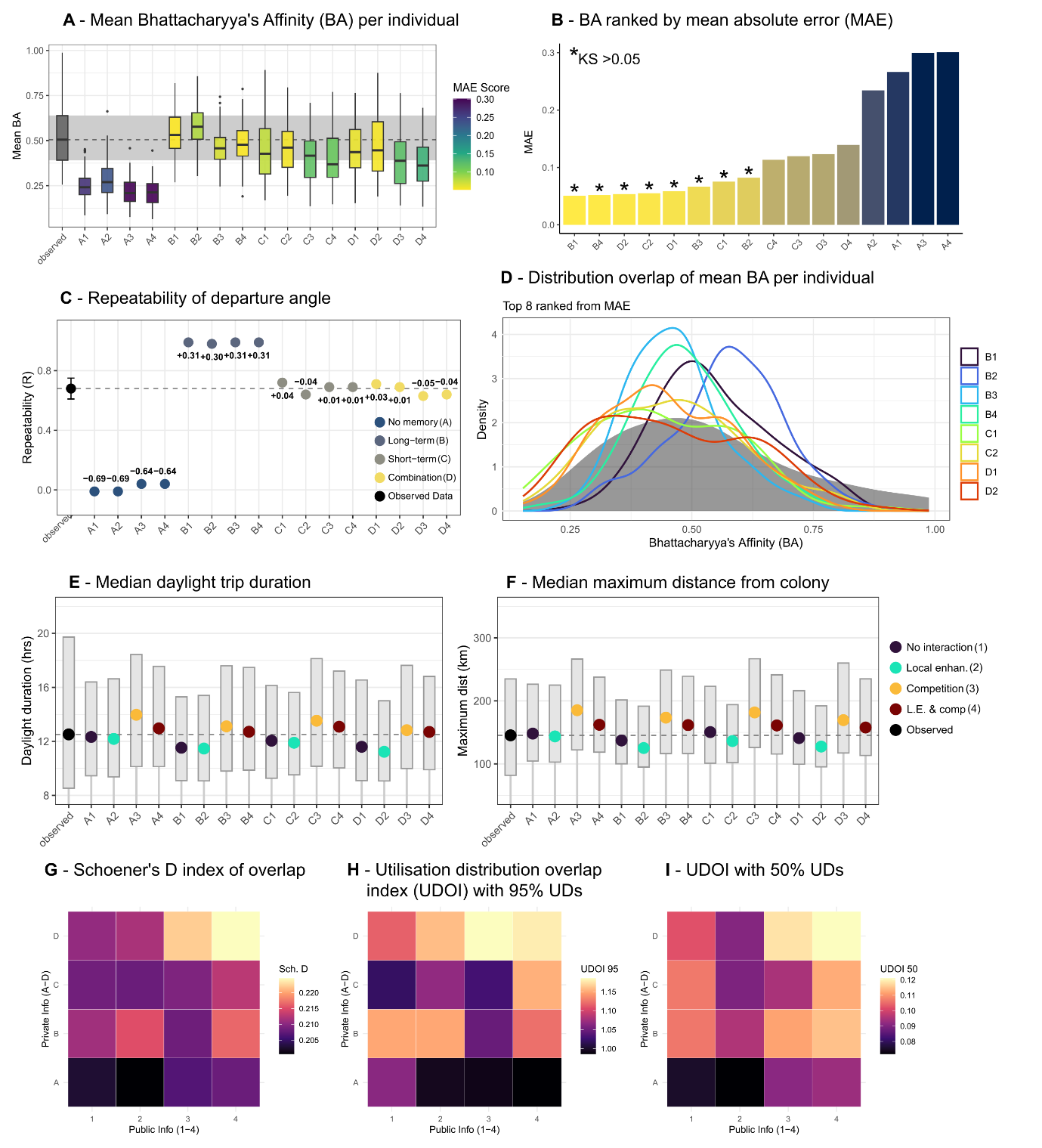


Figure S17: Comparison of simulated movement models against observed data for six consecutive foraging trips per individual. (A-B) Model performance based on Bhattacharyya’s Affinity (BA) and ranked by Mean Absolute Error (MAE), where lower MAE indicates a better fit to observed data. Horizontal grey band in (A) represent the interquartile range of mean BA per individual for observed data (n=33, simulated n=100). Bars marked with an asterisk in B show Kolmogorov-Smirnov (KS) test results for density distributions of simulation vs observed BA where the null hypothesis that the distribution of the simulation is identical to the observed distribution is accepted. (C) Repeatability of departure angles with simulation points coloured by different memory mechanisms and black for observed data. (D) Density distribution of mean BA per individual for the top 8 performing models (see B) compared to observed data. (E-F) Comparison of median daylight trip duration and maximum distance from the colony with colours of points representing different social interaction mechanisms and black for observed data. (G-I) Spatial overlap metrics comparing analysed trips to the putative prey distribution including Schoener’s D and Utilisation Distribution Overlap Index (UDOI) at 95% and 50% levels, plotted against varying mechanisms for use of private and public information.


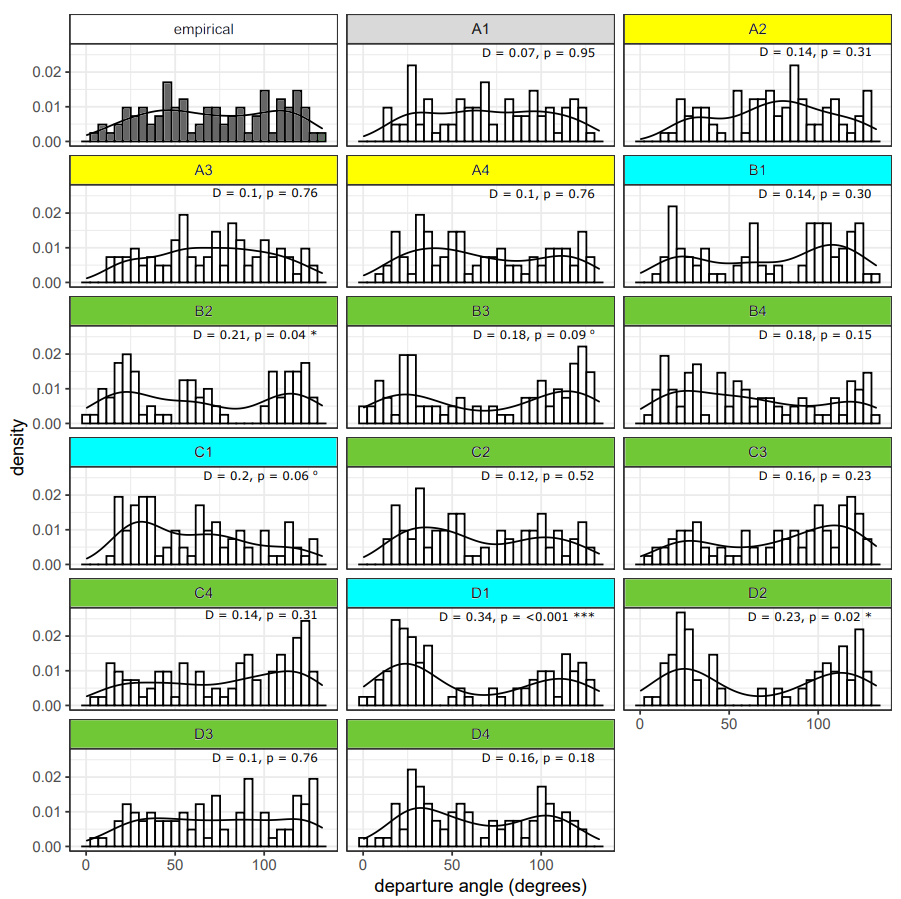


Figure S18: Histograms displaying the distribution of departure angles for empirical data (top left) and the different simulations, with colour representing the different sources of information used in different hypotheses; grey = no information, yellow = public information, blue = private information, green = combination of public and private information. Test statistics in the top right of each panel are from two-sample Kolmogorov-Smirnov tests with the null hypothesis being no difference between the respective simulated distribution and the distribution from empirical data.


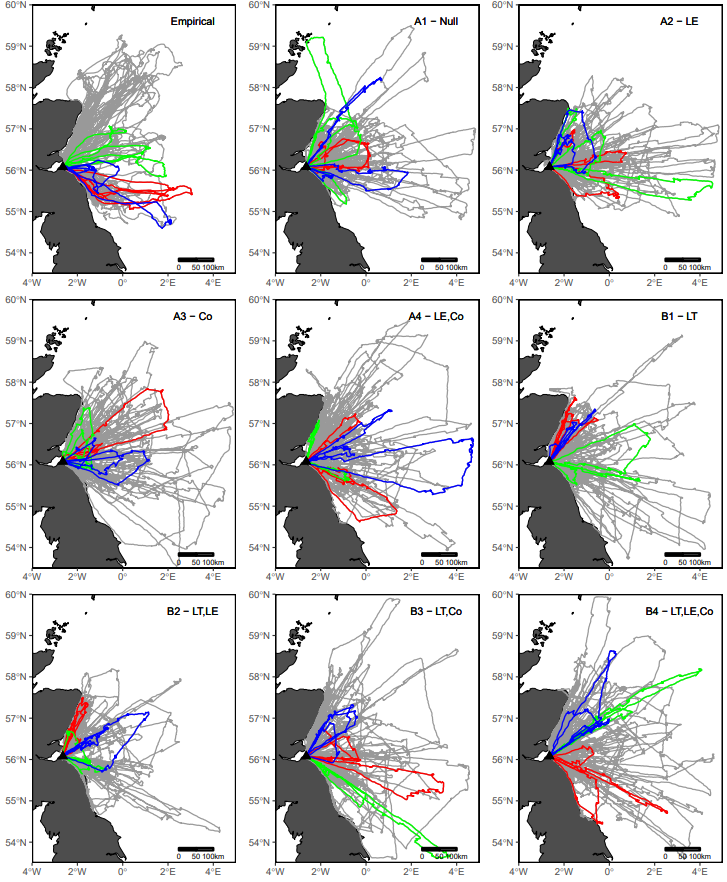


Figure S19: Maps displaying tracking data of 90 trips from 30 individuals (grey) with blue, green, and red paths highlighting three random individuals with three tracks each. The top left is from our empirical data subset, and from top middle, going left to right and top to bottom displays different simulations (A1 – B4) with the hypothesis code and its conditions in the top right. Shorthand for different hypotheses implemented in these simulations in the order they appear: Null = no information available; LE = local enhancement; Co = competition from conspecifics; LT = long-term memory.


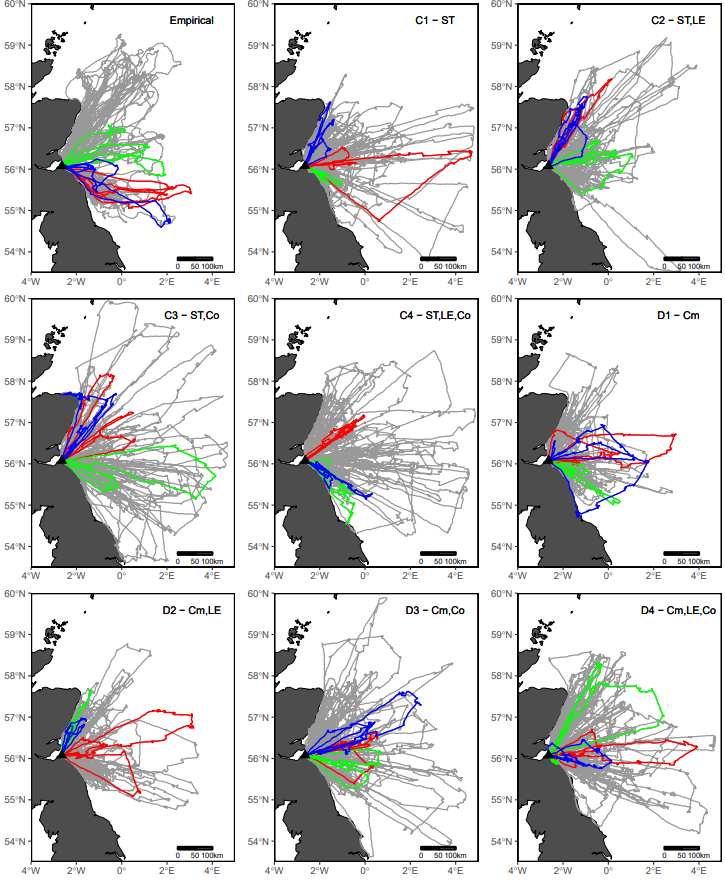


Figure S20: Maps displaying tracking data of 90 trips from 30 individuals (grey) with blue, green, and red paths highlighting three random individuals with three tracks each. The top left is from our empirical data subset, and from top middle, going left to right and top to bottom displays different simulations (C1 – D4) with the hypothesis code and its conditions in the top right. Shorthand for different hypotheses implemented in these simulations in the order they appear: ST = short-term memory; LE = local enhancement; Co = competition from conspecifics; Cm = combined memory.


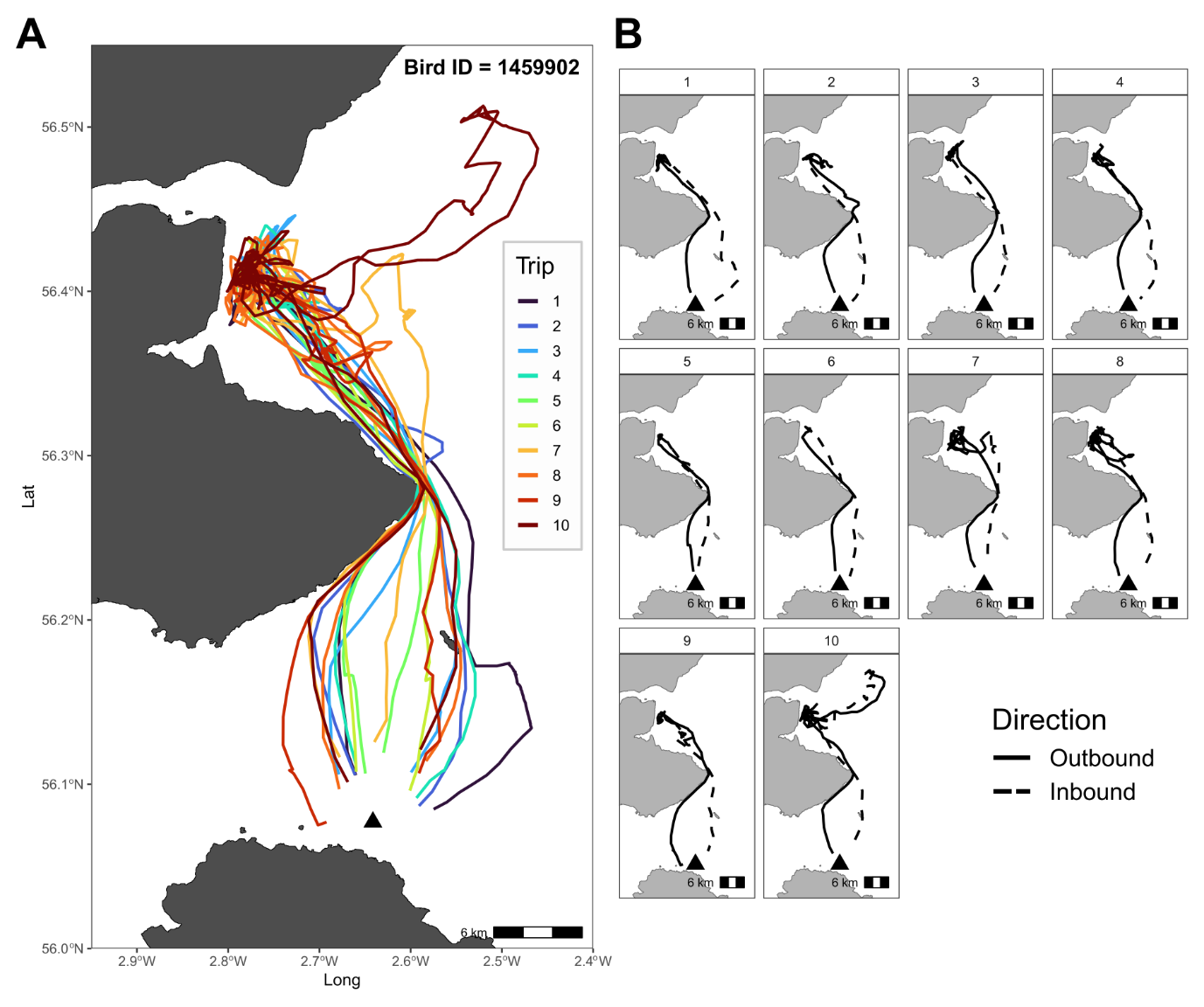


Figure S21: Emprical tracks of one gannet exhibiting very high foraging site fidelity in 2011. Panel A shows ten consecutive trips, with foraging areas off the coast of Tentsmuir Forest in Fife, and panel B displaying each of trips plotted separately with “outbound” and “inbound” legs deduced from time before or after the midpoint each trip’s duration. The black triangle in all plots indicates the location of the colony.

# 9 Empirical support for modelling decisions

Table S9: A table summarising overnight against same day trips for all tracking data available across years of study.

| **Year** | **Total trips** | **N Overnight trips** | **N Same day trips** | **Proportion overnight** | **Proportion same day** |
| --- | --- | --- | --- | --- | --- |
| 2011 | 139 | 111 | 28 | 0.80 | 0.20 |
| 2012 | 149 | 125 | 24 | 0.84 | 0.16 |
| 2015 | 123 | 113 | 10 | 0.92 | 0.08 |
| 2016 | 111 | 84 | 27 | 0.76 | 0.24 |
| All years | 522 | 433 | 89 | 0.83 | 0.17 |


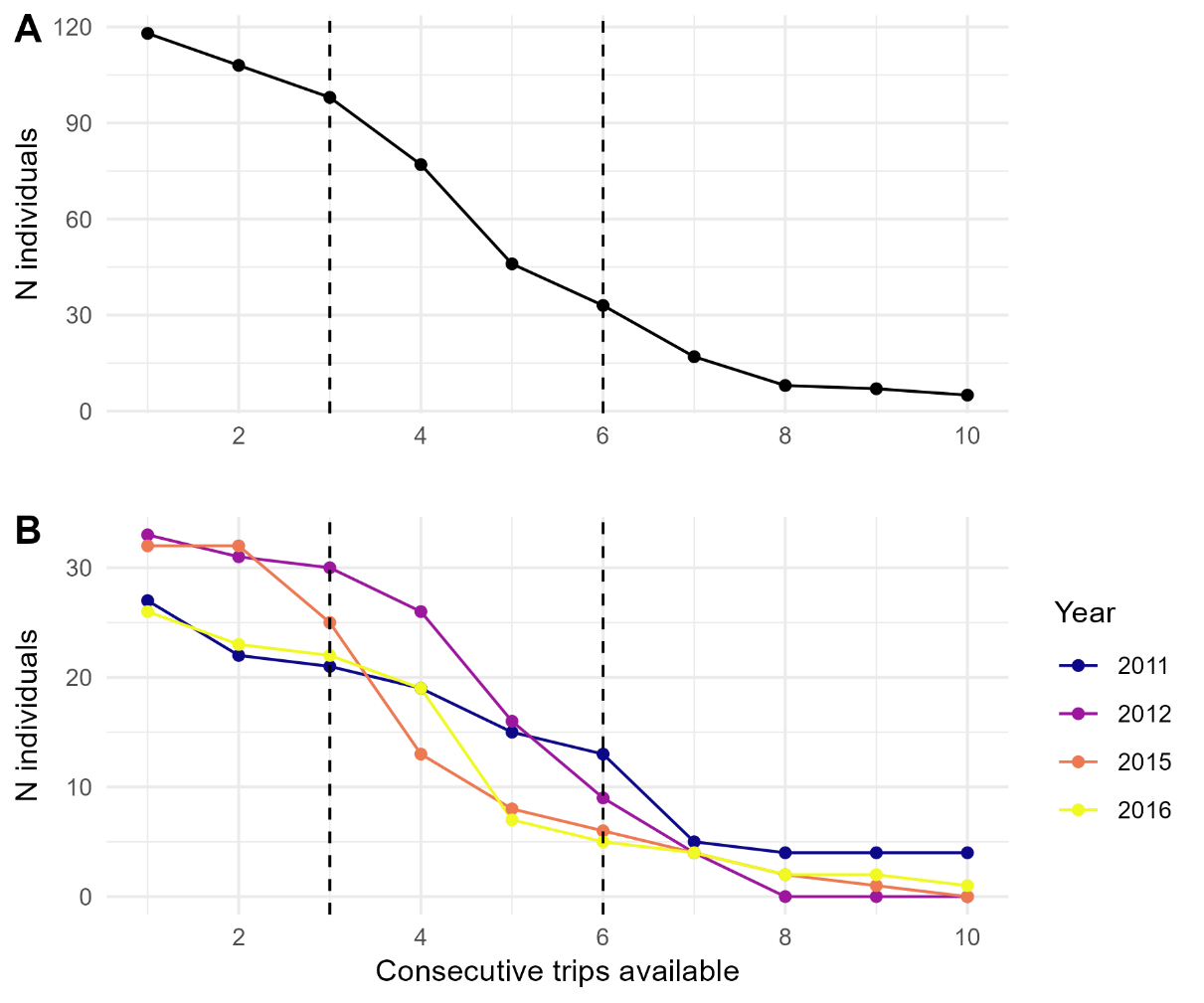


Figure S22: Plot showing the sample size of individuals attainable for a given number of consecutive trips for (A) all years of data combined, and (B) the different years of study plotted separately. Vertical dashed lines indicate the number of consecutive trips used in our study of individual foraging site fidelity patterns.

# References

Ashmole, N. P., Farner, D. S., & King, J. R. (1971). Seabird ecology and the marine environment. *Avian Biology*, *1*, 223–286.

Becker, W. A., & Others. (1975). Manual of quantitative genetics. *Manual of Quantitative Genetics.*, *Ed. 3*. https://www.cabdirect.org/cabdirect/abstract/19750115377

Calenge, C., & Calenge, M. C. (2018). *Package ‘adehabitatLT.’* https://cran.opencpu.org/web/packages/adehabitatLT/adehabitatLT.pdf

Camphuysen. (2011). Northern Gannets in the North Sea: foraging distribution and feeding techniques around the Bass Rock. *British Birds*. https://britishbirds.co.uk/wp-content/uploads/2014/05/V104_N02_P060%E2%80%93076_A.pdf

Carter, M. I. D., Cox, S. L., Scales, K. L., Bicknell, A. W. J., Nicholson, M. D., Atkins, K. M., Morgan, G., Morgan, L., Grecian, W. J., Patrick, S. C., & Votier, S. C. (2016). GPS tracking reveals rafting behaviour of Northern Gannets (Morus bassanus): implications for foraging ecology and conservation. *Bird Study: The Journal of the British Trust for Ornithology*, *63*(1), 83–95.

Enstipp, M. R., Grémillet, D., & Jones, D. R. (2007). Investigating the functional link between prey abundance and seabird predatory performance. *Marine Ecology Progress Series*, *331*, 267–279.

Fieberg, J., & Kochanny, C. O. (2005). Quantifying home-range overlap: The importance of the utilization distribution. *The Journal of Wildlife Management*, *69*(4), 1346–1359.

Furness, R. W., Garthe, S., Trinder, M., Matthiopoulos, J., Wanless, S., & Jeglinski, J. (2018). Nocturnal flight activity of northern gannets Morus bassanus and implications for modelling collision risk at offshore wind farms. *Environmental Impact Assessment Review*, *73*, 1–6.

Garthe, S., Grémillet, D., & Furness, R. W. (1999). At-sea-activity and foraging efficiency in chick-rearing northern gannets Sula bassana: a case study in Shetland. *Marine Ecology Progress Series*, *185*, 93–99.

Grecian, W. J., Lane, J. V., Michelot, T., Wade, H. M., & Hamer, K. C. (2018). Understanding the ontogeny of foraging behaviour: insights from combining marine predator bio-logging with satellite-derived oceanography in hidden Markov models. *Journal of the Royal Society, Interface*, *15*(143), 20180084.

Grimm, V., Berger, U., Bastiansen, F., Eliassen, S., Ginot, V., Giske, J., Goss-Custard, J., Grand, T., Heinz, S. K., Huse, G., Huth, A., Jepsen, J. U., Jørgensen, C., Mooij, W. M., Müller, B., Pe’er, G., Piou, C., Railsback, S. F., Robbins, A. M., … DeAngelis, D. L. (2006). A standard protocol for describing individual-based and agent-based models. *Ecological Modelling*, *198*(1), 115–126.

Grimm, V., & Railsback, S. F. (2012). Pattern-oriented modelling: a “multi-scope” for predictive systems ecology. *Philosophical Transactions of the Royal Society of London. Series B, Biological Sciences*, *367*(1586), 298–310.

Grimm, V., Railsback, S. F., Vincenot, C. E., Berger, U., Gallagher, C., Deangelis, D. L., Edmonds, B., Ge, J., Giske, J., Groeneveld, J., Johnston, A. S. A., Milles, A., Nabe-Nielsen, J., Polhill, J. G., Radchuk, V., Rohwäder, M. S., Stillman, R. A., Thiele, J. C., & Ayllón, D. (2020). The ODD protocol for describing agent-based and other simulation models: A second update to improve clarity, replication, and structural realism. *Journal of Artificial Organs: The Official Journal of the Japanese Society for Artificial Organs*, *23*(2). http://eprints.bournemouth.ac.uk/33918/

Hamer, K. C., Humphreys, E. M., Garthe, S., Hennicke, J., Peters, G., Grémillet, D., Phillips, R. A., Harris, M. P., & Wanless, S. (2007). Annual variation in diets, feeding locations and foraging behaviour of gannets in the North Sea: flexibility, consistency and constraint. *Marine Ecology Progress Series*, *338*, 295–305.

Hamer, K. C., Humphreys, E. M., Magalhães, M. C., Garthe, S., Hennicke, J., Peters, G., Grémillet, D., Skov, H., & Wanless, S. (2009). Fine-scale foraging behaviour of a medium-ranging marine predator. *The Journal of Animal Ecology*, *78*(4), 880–889.

Hamer, K. C., Phillips, R. A., Hill, J. K., Wanless, S., & Wood, A. G. (2001). Contrasting foraging strategies of gannets Morus bassanus at two North Atlantic colonies: foraging trip duration and foraging area fidelity. *Marine Ecology Progress Series*, *224*, 283–290.

Hamer, K. C., Phillips, R. A., Wanless, S., Harris, M. P., & Wood, A. G. (2000). Foraging ranges, diets and feeding locations of gannets Morus bassanus in the North Sea: evidence from satellite telemetry. *Marine Ecology Progress Series*, *200*, 257–264.

Holling, C. S. (1959). Some Characteristics of Simple Types of Predation and Parasitism1. *The Canadian Entomologist*, *91*(7), 385–398.

Jones, T. B., Patrick, S. C., Arnould, J. P. Y., Rodríguez-Malagón, M. A., Wells, M. R., & Green, J. A. (2018). Evidence of sociality in the timing and location of foraging in a colonial seabird. *Biology Letters*, *14*(7), 20180214.

Kamil, A. C. (1983). Optimal foraging theory and the psychology of learning. *American Zoologist*, *23*(2), 291–302.

Lane, J. V., Jeavons, R., Deakin, Z., Sherley, R. B., Pollock, C. J., Wanless, R. J., & Hamer, K. C. (2020). Vulnerability of northern gannets to offshore wind farms; seasonal and sex-specific collision risk and demographic consequences. *Marine Environmental Research*, *162*, 105196.

Lane, J. V., Jeglinski, J. W. E., Avery-Gomm, S., Ballstaedt, E., Banyard, A. C., Barychka, T., Brown, I. H., Brugger, B., Burt, T. V., Careen, N., Castenschiold, J. H. F., Christensen-Dalsgaard, S., Clifford, S., Collins, S. M., Cunningham, E., Danielsen, J., Daunt, F., D’entremont, K. J. N., Doiron, P., … Votier, S. C. (2023). High pathogenicity avian influenza (H5N1) in Northern Gannets (*Morus bassanus*): Global spread, clinical signs and demographic consequences. *The Ibis*, *166*(2), 633–650.

Lane, J. V., Spracklen, D. V., & Hamer, K. C. (2019). Effects of windscape on three-dimensional foraging behaviour in a wide-ranging marine predator, the northern gannet. *Marine Ecology Progress Series*, *628*, 183–193.

Lessells, C. M., & Boag, P. T. (1987). Unrepeatable Repeatabilities: A Common Mistake. *The Auk*, *104*(1), 116–121.

Lewis, S., Sherratt, T. N., Hamer, K. C., & Wanless, S. (2001). Evidence of intra-specific competition for food in a pelagic seabird. *Nature*, *412*(6849), 816–819.

Liukkonen, L., Ayllón, D., Kunnasranta, M., Niemi, M., Nabe-Nielsen, J., Grimm, V., & Nyman, A.-M. (2018). Modelling movements of Saimaa ringed seals using an individual-based approach. *Ecological Modelling*, *368*, 321–335.

Murray, S., Harris, M. P., & Wanless, S. (2015). The status of the Gannet in Scotland in 2013-14. *Scottish Birds*, *35*(1). http://nora.nerc.ac.uk/510050/1/N510050JA.pdf

Nelson, B. (2010). *The Gannet*. A&C Black.

Patrick, S. C., Bearhop, S., Grémillet, D., Lescroël, A., Grecian, W. J., Bodey, T. W., Hamer, K. C., Wakefield, E., Le Nuz, M., & Votier, S. C. (2014). Individual differences in searching behaviour and spatial foraging consistency in a central place marine predator. *Oikos* , *123*(1), 33–40.

Pettex, E., Bonadonna, F., Enstipp, M. R., Siorat, F., & Grémillet, D. (2010). Northern gannets anticipate the spatio–temporal occurrence of their prey. *The Journal of Experimental Biology*, *213*(14), 2365–2371.

Ropert-Coudert, Y., Grémillet, D., Kato, A., Ryan, P. G., Naito, Y., & Le Maho, Y. (2004). A fine-scale time budget of Cape gannets provides insights into the foraging strategies of coastal seabirds. *Animal Behaviour*, *67*(5), 985–992.

Soanes, L. M., Arnould, J. P. Y., Dodd, S. G., Sumner, M. D., & Green, J. A. (2013). How many seabirds do we need to track to define home-range area? *The Journal of Applied Ecology*, *50*(3), 671–679.

Soanes, L. M., Atkinson, P. W., Gauvain, R. D., & Green, J. A. (2013). Individual consistency in the foraging behaviour of Northern Gannets: Implications for interactions with offshore renewable energy developments. *Marine Policy*, *38*, 507–514.

Stoffel, M. A., Nakagawa, S., & Schielzeth, H. (2017). rptR: repeatability estimation and variance decomposition by generalized linear mixed‐effects models. *Methods in Ecology and Evolution / British Ecological Society*, *8*(11), 1639–1644.

Sumner, M. D. (2016). *Trip: Tools for the Analysis of Animal Track Data* (Version 1.5.0) [R Package]. https://github.com/mdsumner/trip

Thiebault, A., Mullers, R. H. E., Pistorius, P. A., & Tremblay, Y. (2014). Local enhancement in a seabird: reaction distances and foraging consequence of predator aggregations. *Behavioral Ecology: Official Journal of the International Society for Behavioral Ecology*, *25*(6), 1302–1310.

Tremblay, Y., Thiebault, A., Mullers, R., & Pistorius, P. (2014). Bird-borne video-cameras show that seabird movement patterns relate to previously unrevealed proximate environment, not prey. *PloS One*, *9*(2), e88424.

Votier, S. C., Fayet, A. L., Bearhop, S., Bodey, T. W., Clark, B. L., Grecian, J., Guilford, T., Hamer, K. C., Jeglinski, J. W. E., Morgan, G., Wakefield, E., & Patrick, S. C. (2017). Effects of age and reproductive status on individual foraging site fidelity in a long-lived marine predator. *Proceedings. Biological Sciences / The Royal Society*, *284*(1859). https://doi.org/10.1098/rspb.2017.1068

Wakefield, E. D., Bodey, T. W., Bearhop, S., Blackburn, J., Colhoun, K., Davies, R., Dwyer, R. G., Green, J. A., Grémillet, D., Jackson, A. L., Jessopp, M. J., Kane, A., Langston, R. H. W., Lescroël, A., Murray, S., Le Nuz, M., Patrick, S. C., Péron, C., Soanes, L. M., … Hamer, K. C. (2013). Space partitioning without territoriality in gannets. *Science*, *341*(6141), 68–70.

Wakefield, E. D., Cleasby, I. R., Bearhop, S., Bodey, T. W., Davies, R. D., Miller, P. I., Newton, J., Votier, S. C., & Hamer, K. C. (2015). Long-term individual foraging site fidelity—why some gannets don’t change their spots. *Ecology*, *96*(11), 3058–3074.

Wanless, S., Harris, M. P., & Morris, J. A. (1990). A Comparison of Feeding Areas Used by Individual Common Murres (Uria aalge), Razorbills (Alca torda) and an Atlantic Puffin (Fratercula arctica) during the Breeding Season. *Colonial Waterbirds*, *13*(1), 16–24.

Ward, P., & Zahavi, A. (1973). The importance of certain assemblages of birds as “information‐centres” for food‐finding. *The Ibis*. https://onlinelibrary.wiley.com/doi/abs/10.1111/j.1474-919X.1973.tb01990.x?casa_token=umFu2fiMdU4AAAAA:c7nodjnPWyieJH2I_SxajnNe-v0NsLGADOp1TVKeQoYfSi6YCNTariHCNcDqwkM6QBsTrarhMmQF

Wilensky, U. (1999). *NetLogo*. Northwestern University. http://www.citeulike.org/group/2050/article/1283125

Zurell, D., Berger, U., Cabral, J. S., Jeltsch, F., Meynard, C. N., Münkemüller, T., Nehrbass, N., Pagel, J., Reineking, B., Schröder, B., & Grimm, V. (2010). The virtual ecologist approach: simulating data and observers. *Oikos* , *119*(4), 622–635.

R Core Team (2021) R: A Language and Environment for Statistical Computing. R Foundation for Statistical Computing, Vienna.
https://www.R-project.org
